# Formulation Studies on Microemulsion-Based Polymer Gels Loaded with Voriconazole for the Treatment of Skin Mycoses

**DOI:** 10.3390/pharmaceutics17091218

**Published:** 2025-09-18

**Authors:** Michał Gackowski, Anna Froelich, Oliwia Kordyl, Jolanta Długaszewska, Dorota Kamińska, Raphaël Schneider, Tomasz Osmałek

**Affiliations:** 1 Poznan University of Medical Sciences, Chair and Department of Pharmaceutical Technology, 3 Rokietnicka, 60-806 Poznan, Poland; tosmalek@ump.edu.pl; 2 Poznan University of Medical Sciences, Doctoral School, 70 Bukowska Street, 60-812 Poznan, Poland; 3 Université de Lorraine, CNRS, LRGP, F-54000 Nancy, France; raphael.schneider@univ-lorraine.fr; 4 Poznan University of Medical Sciences, Chair and Department of Pharmaceutical Technology, 3D Printing Division, 3 Rokietnicka, 60-806 Poznan, Poland; froelich@ump.edu.pl (A.F.); okordyl@ump.edu.pl (O.K.); 5 Poznan University of Medical Sciences, Chair and Department of Genetics and Pharmaceutical Microbiology, 3 Rokietnicka, 60-806 Poznan, Poland; jdlugasz@ump.edu.pl (J.D.); dorotakaminska@ump.edu.pl (D.K.)

**Keywords:** voriconazole, microemulsion-based gel, menthol, skin drug delivery, topical antifungal formulation, *Candida albicans*

## Abstract

**Background:** Skin mycoses affect approximately 10% of the global population, and the range of effective topical antifungal agents remains limited. Voriconazole (VRC) is a broad-spectrum triazole with proven efficacy against drug-resistant fungal infections. This study aimed to develop and optimize VRC-loaded microemulsion (ME) polymer gels (Carbopol^®^-based) for cutaneous delivery. Selected formulations also contained menthol (2%) as a penetration enhancer and potential synergistic antifungal agent. **Methods:** A comprehensive screening was performed using pseudoternary phase diagrams to identify stable oil/surfactant/co-surfactant/water systems. Selected MEs were prepared with triacetin, Etocas™ 35, and Transcutol^®^, then gelled with Carbopol^®^. Formulations were characterized for pH, droplet size, polydispersity index (PDI), and viscosity. In vitro VRC release was assessed using diffusion cells, while ex vivo permeation and skin deposition studies were conducted on full-thickness human skin. Rheological behavior (flow curves, yield stress) and texture (spreadability) were evaluated. Antifungal activity was tested against standard strain of *Candida albicans* and clinical isolates including a fluconazole-resistant strain. **Results:** The optimized ME (pH ≈ 5.2; droplet size ≈ 2.8 nm) was clear and stable with both VRC and menthol. Gelation produced non-Newtonian, shear-thinning hydrogels with low thixotropy, favorable for topical application. In ex vivo studies, performed with human skin, both VRC-loaded gels deposited the drug in the epidermis and dermis, with no detectable amounts in the receptor phase after 24 h, indicating retention within the skin. Menthol increased VRC deposition. Antifungal testing showed that VRC-containing gels produced large inhibition zones against *C. albicans*, including the resistant isolate. The VRC–menthol gel exhibited significantly greater inhibition zones than the VRC-only gel, confirming synergistic activity. **Conclusions:** ME-based hydrogels effectively delivered VRC into the skin. Menthol enhanced drug deposition and demonstrated synergistic antifungal activity with voriconazole.

## 1. Introduction

Dermatophytosis, both superficial and deep, remains a significant and often underestimated clinical problem. According to data from the Global Burden of Disease database, approximately 10% of the global population suffers from skin mycoses, making them one of the most common fungal infections worldwide [1]. In April 2025, the World Health Organization (WHO) issued a series of reports drawing attention to the critical shortage of effective antifungal treatments for invasive fungal infections, underscoring the urgent need for innovative research and development (R&D) initiatives to address this growing gap. The reports identified fungal diseases as an escalating public health concern, with widespread infections, such as those caused by *Candida*, responsible for oral and vaginal thrush, increasingly resistant to the existing therapeutic options. Particularly concerning are the fungal pathogens listed in the “critical priority” group of the WHO Fungal Priority Pathogens List (FPPL), associated with mortality rates reaching up to 88%. The WHO emphasized the necessity of developing safer, more effective antifungal agents that could reduce both treatment-related toxicity and the burden of intensive patient monitoring [2]. Adding to this challenge, another WHO report on antimicrobial resistance highlights the growing prevalence of drug-resistant fungal infections, which increasingly contribute to treatment failures, prolonged hospitalizations, and significantly higher healthcare costs [3,4]. In light of these alarming trends, there is a clear and pressing need to develop more effective and, above all, safer antifungal treatment strategies capable of overcoming current therapeutic limitations and addressing the evolving threat of antifungal resistance.

A population-based study assessing common skin diseases in Europe shows that fungal infections account for 8.9% of all skin issues, ranking first among reported infections [5]. Reports indicate that most of these infections are caused by *Candida* spp., but other pathogens such as *Malassezia* spp., *Aspergillus* spp., dimorphic fungi, and *Mucorales* spp. also contribute to the problem [6]. Treating dermatophytoses, especially those affecting deeper skin layers, remains time-consuming and typically requires systemic antifungal therapy. However, prolonged oral antifungal use carries the risk of serious adverse effects, which is why topical antifungal agents are frequently employed as safer alternatives [7]. Unfortunately, the range of available topical antifungals is relatively limited, with clotrimazole, econazole, miconazole, ketoconazole, terbinafine, nystatin, and ciclopirox representing the most commonly used compounds [8].

As a significant challenge in treating mycoses is the growing resistance of microorganisms to existing drugs, there is considerable optimism regarding developing new drug delivery systems that can ensure both therapy safety and maximum therapeutic effect. Clinical data demonstrate the exceptional effectiveness of topical VRC, particularly for difficult-to-treat infections, though experimental studies on its delivery systems are still limited [9]. VRC shows clear advantages in treating drug-resistant infections [10,11]. Given the alarming data indicating that a significant portion of the population suffers from skin fungal infections, VRC was chosen as the active compound. It should be emphasized that despite the promising results of preliminary studies describing topical applications of VRC, no commercial product has been introduced to the pharmaceutical market so far.

VRC is a second-generation triazole antifungal agent structurally related to fluconazole but distinguished by its broader spectrum of activity. It is effective against a wide range of opportunistic fungal pathogens, including yeasts, molds, and dimorphic fungi. Like other triazoles, VRC exerts its antifungal effect by inhibiting the cytochrome P450-dependent enzyme responsible for converting lanosterol to ergosterol, a key component of the fungal cell membrane. This inhibition leads to ergosterol depletion, accumulation of toxic sterol intermediates, and ultimately the disruption of membrane integrity and fungal cell death. Unlike fluconazole, whose activity is limited to yeasts, VRC exhibits broad-spectrum efficacy against both yeasts and molds, making it a versatile agent for the treatment of superficial and invasive fungal infections [12].

One of the major obstacles in the effective topical delivery of pharmacologically active compounds is the inherently low permeability of the skin, which serves as a robust physiological barrier. In particular, the stratum corneum, the outermost layer of the skin, plays a key role in limiting the penetration of exogenous substances into the underlying tissues. To overcome this limitation, various formulation strategies have been developed, many of which focus on the use of advanced drug delivery systems. Among these, nanocarrier-based approaches have garnered significant attention, including submicron emulsions [13,14]. Microemulsions (MEs), a subclass of submicron systems, are thermodynamically stable, isotropic systems characterized by their transparent appearance and extremely small droplet size, typically ranging from 10 to 100 nm. Their nanometric scale is responsible for the lack of light scattering, which contributes to their clear appearance [15]. Unlike macroemulsions, MEs form spontaneously upon mixing their components, without the need for high-energy input, provided the appropriate composition is maintained. This spontaneous formation is a defining feature that sets MEs apart from nanoemulsions, which generally require mechanical energy for droplet size reduction due to their lower surfactant content. Importantly, MEs maintain their stability unless disrupted by significant changes in temperature or phase ratios, making them attractive candidates for drug delivery systems [16,17]. Studies have consistently demonstrated that MEs can significantly improve drug permeation through the stratum corneum, potentially leading to enhanced therapeutic outcomes. Furthermore, their biphasic nature allows for the simultaneous incorporation of both hydrophilic and lipophilic substances, including permeation enhancers, thereby increasing formulation versatility. However, due to their inherently low viscosity, MEs in liquid form may pose practical limitations for topical use [18]. To address this, they are often transformed into semisolid systems, such as gels, which improve their mechanical properties and user acceptability.

In this study, a comprehensive development and evaluation of ME-based gel formulations containing VRC as the active pharmaceutical ingredient (API) was undertaken. Initially, six different oil phases, five surfactants, and two co-surfactants were screened, resulting in the construction of 46 pseudoternary phase diagrams to identify compositions with the greatest stability and broad ME-forming capacity. Solubility studies of VRC in selected components were subsequently performed to support formulation design. Based on the screening results, five MEs varying in oil phase composition were tested for drug release profiles, allowing for the selection of the most promising oil phase for further investigation. This was followed by ex vivo permeation and skin deposition studies using human skin, carried out on three MEs differing in surfactant type. The most effective formulation, selected based on permeability data, underwent detailed physicochemical characterization and was further transformed into a semisolid gel. The resulting ME-based gels were assessed in terms of drug permeation and skin deposition, as well as their rheological and textural properties. In addition, microbiological studies were performed using two clinical *Candida albicans* isolates and the ATCC 10231 reference strain. The potential enhancing effect of menthol was also evaluated with respect to drug permeation, antifungal activity, and mechanical characteristics.

## 2. Materials and Methods

### 2.1. Materials

Triacetin 99%, di(ethylene glycol) ethyl ether (Transcutol^®^) ≥ 99%, polyethylene glycol 400 (PEG 400), Tween^®^ 80, Ludox^®^ HS-30 colloidal silica, ethyl oleate, Tween^®^ 85, isopropyl myristate, isopropyl palmitate, oleic acid, triisopropanolamine 95%, and voriconazole pharmaceutical secondary standard were purchased from Sigma-Aldrich (St. Louis, MO, USA). Neobee^®^ M-5 was obtained from Stepan Company (New York, NY, USA). Brij^®^ O5-SS-(RB) and Etocas^TM^ 35 Pharma-LQ-(RB) were supplied by Croda Europe (Goole, UK). Phosphate-buffered saline (PBS) pH 7.4 concentrate and orthophosphoric acid 85% were purchased from Chempur (Piekary Śląskie, Poland). Sodium chloride pure, acetonitrile HPLC grade, and ethanol absolute 99.8% were obtained from Avantor™ Performance Materials Poland S.A. (Gliwice, Poland). Levomenthol was purchased from Fagron (Kraków, Poland). Carbopol^®^ Ultrez 21 polymer was supplied by Lubrizol (Wickliffe, OH, USA). Voriconazole ≥ 98% was obtained from Acros Organics (Geel, Belgium). Ultrapure water was used in all experiments.

### 2.2. Methodology

**Figure 1:**
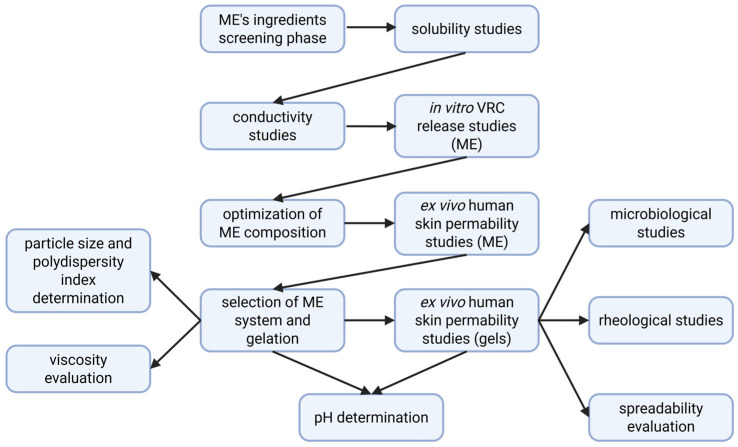
Workflow of formulation development and evaluation of VRC-loaded microemulsion and hydrogel systems.

Figure 1 presents the experimental workflow for the development, optimization, and evaluation of VRC-loaded MEs and their gelled forms, including formulation screening, physicochemical characterization, in vitro release, ex vivo skin permeability, and microbiological and rheological assessments.

#### 2.2.1. Microemulsion Preparation

To identify the ME region, a pseudoternary phase diagram was constructed using the water titration method at 25.0 ± 0.5 °C. In the first step, surfactant and co-surfactant mixtures were prepared in two weight ratios: 1:1 and 1:2. Subsequently, 4.0 g samples containing oil and surfactant/co-surfactant mixtures at various weight ratios (1:9, 2:8, 3:7, 4:6, 5:5, 6:4, 7:3, 8:2, and 9:1) were prepared and titrated with ultrapure water until turbidity was observed. During the titration process, the samples were gently mixed and visually inspected. Compositions that resulted in transparent, monophasic liquids were classified as MEs, whereas turbid systems were assigned to the coarse emulsion category. The systems that were the subject of more detailed investigations in this study are presented in Table 1, while all systems constructed within the research are listed in Table S1 (Supplementary Material).

#### 2.2.2. Solubility Studies of Voriconazole

The solubility of VRC in selected solvents was evaluated using the Crystal 16^®^ Crystallization System (Avantium Technologies, Amsterdam, The Netherlands), equipped with Crystal16™ software (v1.14.0). Data analysis was performed using the Crystal Clear™ software (v1.0, Avantium). The Crystal 16 system monitors solubility behavior based on turbidity measurements using in situ light transmission. A laser beam is passed through each vial, and the amount of transmitted light is recorded in real time. A sharp decrease in transmission indicates the onset of precipitation, whereas a sudden increase reflects complete dissolution of the solute. This allows precise determination of solubility limits as a function of temperature.

For each solvent, four solutions with increasing concentrations of VRC were prepared using a fixed solvent volume of 1.0 mL. The experiments were conducted in three consecutive temperature cycles. Initially, a baseline signal was recorded for each pure solvent. Next, solubility measurements of VRC were performed under controlled temperature conditions. The system operated within a temperature range of 0 °C to 60 °C, with a bottom stirring rate of 800 rpm. In the first cycle, the experiment was initiated at 10 °C (or 20 °C for solvents with a higher freezing point). The temperature was gradually increased to 60 °C at a rate of 0.5 °C/min and then held at 60 °C for 10 min to ensure complete dissolution. Subsequently, the temperature was decreased to 0 °C at a rate of −0.5 °C/min. The second and third heating–cooling cycles were performed between 0 °C and 60 °C under the same heating and cooling rates.

#### 2.2.3. Electrical Conductivity Studies

Electrical conductivity measurements were carried out to evaluate the internal structure and phase behavior of selected MEs. The experiments were conducted at 25.0 ± 0.5 °C using a FiveEasy™ conductivity meter (FE30; Mettler Toledo, Greifensee, Switzerland), calibrated with standard solutions of 1413 μS/cm and 12.88 mS/cm to ensure measurement precision. All measurements were performed in triplicate, and the results were expressed as mean values with standard deviations (SD). The analyzed formulations (10.0 g) consisted of the oil phase and a surfactant/co-surfactant mixture (1:1 *w*/*w*) in weight ratios of 4:6, 3:7, and 2:8. Each system was titrated stepwise with a 0.01% (*w*/*v*) sodium chloride aqueous solution. After each addition, the sample was gently stirred to ensure homogeneity, and conductivity was recorded. The titration continued until turbidity was observed. These measurements enabled the differentiation between water-in-oil, bicontinuous, and oil-in-water structures based on conductivity profiles. Importantly, the results of the conductivity studies also guided the selection of optimal water content for further formulation development. Initial screening was performed on systems A_9.1_, A_13.1_, A_16.1_, A_19.1_, and A_23.1_ (Table 1). In a subsequent phase of the study, additional measurements were conducted for systems A_2.1_, A_4.1_, and A_9.1_ formulated at an oil phase-to-surfactant/co-surfactant mixture (1:1 *w*/*w*) ratio of 3:7 (*w*/*w*).

#### 2.2.4. Preparation of Drug-Loaded Microemulsions

MEs selected for further investigation were prepared by mixing the oil phase with a surfactant/co-surfactant mixture (1:1 *w*/*w*). Solid components (VRC and/or menthol) were then added and dissolved in the resulting solution. In the final step, water was introduced to the system. The formulation was gently stirred until a clear, monophasic liquid was obtained, confirming the formation of a stable ME.

#### 2.2.5. pH Measurements

The pH values of the MEs and the corresponding polymer-based hydrogels were measured using a CG 842 Schott pH-meter (Schott Instruments GmbH, Weilheim, Germany) equipped with a SenTix^®^ Sp-DIN probe (WTW, Pomiarowy i Analityczny Sprzęt Techniczny Sp. z o.o., Wrocław, Poland). All measurements were performed in triplicate, and the results were expressed as average values with standard deviation.

#### 2.2.6. Microemulsion Viscosity Measurements

Viscosity of the MEs was determined using a rotational rheometer HAAKE™ RheoStress 1 (Thermo Electron Corp., Waltham, MA, USA), equipped with a Thermo HAAKE™ DC 30 temperature control unit and Z20 DIN coaxial cylinder geometry (sample volume: 8.2 mL; gap: 4.2 mm). Measurements were carried out at 25.0 ± 0.5 °C in controlled shear rate mode, with the shear rate linearly increasing from 1 to 200 s^−1^. Each analysis was performed in triplicate, and average viscosity values were reported. The shear stress (*τ*) versus shear rate ($\dot{\gamma }$) relationship was analyzed and fitted to the Newtonian flow model.

#### 2.2.7. Particle Size Analysis

Particle size measurements were carried out using the dynamic light scattering (DLS) technique at 25.0 ± 0.5 °C with a Zetasizer Nano ZS instrument (Malvern Instruments Ltd., Worcestershire, UK), equipped with a He-Ne laser (λ = 633 nm) operating in backscattering mode at a detection angle of 173°. Approximately 1 mL of the undiluted ME was transferred into a disposable polystyrene cuvette. Prior to DLS analysis, the refractive index of each sample was determined using a digital handheld refractometer DR201-95 (Kruss Optronic™, Hamburg, Germany), to ensure accurate data interpretation.

#### 2.2.8. Quantification of Voriconazole by HPLC

The concentration of VRC in the collected samples was determined using a validated high-performance liquid chromatography (HPLC) method. Analyses were performed on a UHPLC Nexera-i LC-2040C system (Shimadzu, Kyoto, Japan) equipped with a Luna Omega 5 µm C18 100 Å LC column (250 × 4.6 mm; Phenomenex, Aschaffenburg, Germany), along with SecurityGuard™ C18 cartridges (4 × 3 mm) and a SecurityGuard™ guard cartridge kit (Phenomenex, Aschaffenburg, Germany).

The chromatographic separation was conducted under isocratic conditions using a mobile phase composed of acetonitrile and water (60:40, *v*/*v*) at a flow rate of 1.0 mL/min. The column temperature was maintained at 30.0 °C, detection was performed at 256 nm, and the injection volume was set to 10 μL.

The method was validated in terms of linearity, precision, accuracy, and sensitivity, including both intraday and interday validation. Validation was performed independently by at least two analysts using VRC solutions prepared in phosphate-buffered saline (PBS, pH 7.4) and in absolute ethanol. Calculated parameters are specified in Table S2 (Supplementary Material).

For VRC in PBS, the limit of detection (LOD) was 1.406 ± 0.191 μg/mL, and the limit of quantification (LOQ) was 4.259 ± 0.579 μg/mL. For VRC in absolute ethanol, the LOD was 1.712 ± 0.339 μg/mL, and the LOQ was 5.187 ± 1.027 μg/mL.

#### 2.2.9. In Vitro Release Studies of Voriconazole from Microemulsions

In vitro release of VRC was evaluated from selected MEs (A_9.1_, A_13.1_, A_16.1_, A_19.1_, and A_23.1_), differing in oil phase composition. All formulations were prepared at an oil–surfactant/co-surfactant (1:1 *w*/*w*) ratio of 3:7, with a fixed water content of 23% (*w*/*w*). The experiments were performed using Franz diffusion cells (Teledyne Hanson Research, Chatsworth, CA, USA) with a diffusion area of 1 cm^2^. The donor chamber was loaded with 0.5 g of formulation, and the receptor compartment (volume: 9.8 mL) was filled with phosphate buffer (pH 7.4) containing 10% (*v*/*v*) absolute ethanol to maintain sink conditions.

Diffusion was carried out across a SnakeSkin™ dialysis membrane (MWCO 10 kDa, 35 mm dry I.D.; Thermo Scientific™, Rockford, IL, USA). The temperature was maintained at 32.0 ± 0.5 °C to simulate skin surface conditions, with continuous stirring at 200 rpm. At predetermined time intervals (10, 30, 60, 120, 180, 360, and 720 min), 0.4 mL of receptor medium was withdrawn into HPLC vials and immediately replaced with an equal volume of fresh medium to maintain a constant volume throughout the experiment. All tests were performed in six replicates, and the results were expressed as mean values ± standard deviation (SD).

The concentration of VRC in the samples was determined using a validated HPLC method. Based on the determined drug concentrations, the cumulative amount of VRC that permeated into the acceptor compartment at each time point was calculated using Equation (1):

(1) $$Q=\;\frac{C_{n}\cdot V\;+\;\sum_{i=1}^{n-1}C_{i}\;\cdot S}{A}$$

where *Q* is the cumulative drug amount, *C_n_* is the VRC concentration determined at the *n*th sampling point, *V* is the Franz cell volume, $\sum_{i=1}^{n-1}C_{i}$ is the sum of the drug concentrations determined at timepoints 1 through *n* − 1, *S* is the withdrawn sample volume, and *A* is the effective diffusion area.

#### 2.2.10. Preparation of Voriconazole-Loaded ME-Based Hydrogels

In the first step of hydrogel preparation, VRC was dissolved in a pre-mixed combination of the oil phase and a surfactant/co-surfactant mixture (1:1 *w*/*w*; total ratio 3:7). Subsequently, water was added, and the system was gently stirred until a clear ME was formed. In the next step, Carbopol^®^ Ultrez 21 was gradually dispersed into the system using an overhead stirrer operating at 800 rpm. Once a uniform dispersion was achieved, a pre-prepared solution of triisopropanolamine (TIPA) in water was added to induce gelation, followed by further mixing at 600 rpm until a homogeneous hydrogel was obtained. It should be noted that a portion of the total water content was used to dissolve TIPA and added together with the neutralizing agent to initiate gel formation.

For hydrogels containing menthol, the preparation procedure was slightly modified. Menthol was first dissolved in a small volume of the oil–surfactant/co-surfactant mixture (1:1 *w*/*w*; total ratio 3:7). This menthol-containing solution was added only after the system had been gelled with TIPA and was then mixed thoroughly at 600 rpm to ensure complete incorporation.

It should be noted that the different method of preparation for gels with and without menthol represents a limitation when comparing their rheological and textural properties.

The composition of the prepared ME-based hydrogels is presented in Table 2.

#### 2.2.11. Ex Vivo Skin Permeation and Skin Deposition Studies of Voriconazole

Ex vivo skin permeation and deposition studies of VRC were conducted for selected MEs (A_2.1_, A_4.1_, and A_9.1_), differing in surfactant composition, and ME-based polymer hydrogels (B_2_ and B_3_). All formulations were prepared at an oil–surfactant/co-surfactant (1:1 *w*/*w*) ratio of 3:7, with a fixed water content of 38% (*w*/*w*). Permeation experiments were performed using Franz diffusion cells (Teledyne Hanson Research, Chatsworth, CA, USA) with an effective diffusion area of 1 cm^2^. The donor chamber was loaded with 0.5 g of formulation, and the receptor compartment (volume: 9.6 mL) was filled with phosphate buffer (pH 7.4) containing 10% (*v*/*v*) absolute ethanol to maintain sink conditions.

The permeation study was conducted across full-thickness human skin samples obtained commercially from Biopredic International (Rennes, France). The samples were collected from patients who had undergone surgical operations. The skin samples were stored at −80 °C until use. Prior to the experiment, the skin was equilibrated in phosphate-buffered saline (PBS, pH 7.4) at 32 °C for 20 min and then carefully blotted dry.

All experiments were conducted at 32.0 ± 0.5 °C to mimic physiological skin temperature, with continuous stirring at 200 rpm. At predefined time intervals (30, 60, 120, 180, 360, 540, 720, 1080, and 1440 min), 0.4 mL of receptor medium was withdrawn into HPLC vials and immediately replaced with an equal volume of fresh buffer to maintain a constant receptor volume. Each experiment was performed in triplicate, and results were reported as mean values ± standard deviation (SD).

Following the permeation study, the amount of VRC retained in the skin was also quantified. The skin section that had been in contact with the formulation was washed with water and excised using scissors and a scalpel, then subjected to homogenization and drug extraction. The tissue samples were placed in prefilled tube kits with TriplePure M-bioGrade high-impact zirconium beads (3 mm) (Benchmark Scientific, Sayreville, NJ, USA), combined with a 1:1 (*v*/*v*) mixture of absolute ethanol and water, and subjected to extraction by homogenization performed in three cycles of 180 s each at 3000 rpm using a microtube homogenizer (D1030-E, BeadBug, Benchmark Scientific, Sayreville, NJ, USA). After homogenization, the samples were centrifuged (15,000 rpm, 15 min; IKA G-L, Warszawa, Poland) and the resulting supernatant was collected. The supernatant was filtered through a 0.22 μm nylon syringe filter and analyzed by HPLC, as described in Section 2.2.8. The amount of VRC deposited in the skin was normalized to the weight of the tissue used for homogenization and extraction.

Human skin samples used in the ex vivo permeation and deposition studies were obtained from Biopredic International (Rennes, France) as a commercial product intended exclusively for scientific research. According to the supplier’s certification, Biopredic International strictly complies with ethical rules for donation and collection of human tissues and holds all required regulatory permits for procurement, processing, transfer, and export. Therefore, approval from a bioethics committee was not required for this study.

#### 2.2.12. Determination of Drug Content in Hydrogels

To determine the drug content of VRC in the prepared hydrogels, an accurately weighed 0.1 g sample of each hydrogel was transferred into a 10 mL volumetric flask. The sample was first dispersed in a portion of absolute ethanol, and the volume was then adjusted to 10 mL with the same solvent. Extraction was performed in an ultrasonic water bath (Sonic-6, Polsonic Palczyński Sp.J., Warszawa, Poland) for 20 min. Following extraction, the resulting solutions were filtered using 0.22 μm nylon syringe filters. The filtered samples were then subjected to HPLC-UV analysis as described in Section 2.2.9. Each measurement was performed in triplicate, and results were expressed as mean values ± standard deviation (SD).

#### 2.2.13. Spreadability Test

The spreadability of the hydrogels was assessed using a texture analyzer Shimadzu AGS-X (10 N–10 kN; Shimadzu, Kyoto, Japan) equipped with 90° conical fixtures and operated with TRAPEZIUM X software (version 1.52). The test was conducted at ambient temperature, and each measurement was performed in triplicate (n = 3). A defined amount of hydrogel sample was placed in the lower (female) cone, after which the upper (male) cone was lowered at a speed of 10 mm/min until reaching a depth of 10 mm. It was then withdrawn to a height of −5 mm. The instrument recorded the force required to immerse and separate the cone from the sample, enabling evaluation of the sample’s spreadability characteristics.

#### 2.2.14. Rheological Measurements

Rheological evaluations were performed using a HAAKE^TM^ RheoStress1 rotational rheometer (Thermo Electron Corp., Waltham, MA, USA), with temperature control maintained by a HAAKE^TM^ DC30 thermostat connected to a recirculating water bath. A titanium plate–plate system (diameter 35 mm) was employed for the measurements. After incubating the sample for 10 min at 25 °C, it was carefully placed onto the lower plate using a spatula. The gap between the plates was set to 1.0 mm. Once the upper plate was positioned, any excess sample was gently removed to avoid unintended shear. A waiting time of 120 s was allowed before measurement to enable sample equilibration. The entire procedure was conducted at a constant temperature of 25.0 ± 0.2 °C. Data acquisition and analysis were performed using HAAKE^TM^ RheoWinTM Job Manager and Data Manager software (version 4.91.0021, Thermo Electron Corp.). Each formulation was tested on a fresh sample in triplicate or more, and average parameter values were reported.

##### Steady Shear Experiments—Flow Curves and Thixotropy

Flow behavior was assessed by plotting shear stress (τ) against shear rate ($\dot{\gamma }$). The shear rate was increased from 0 to 300.0 s^−1^ and then decreased back to 0 s^−1^, with each ramp lasting 60 s and no pause between them. Thixotropy was quantified as the relative hysteresis area (Kd), calculated as the ratio of the hysteresis loop area to the total area under the ascending curve.

##### Stress Ramp Test (CS—Controlled Stress)

The stress ramp test was conducted over a shear stress range of 1.0–500.0 Pa, with a total test duration of 60 s. During the measurement, 180 data points were recorded. The results were plotted as strain versus shear stress on a logarithmic scale.

#### 2.2.15. Antifungal Properties

The antifungal activity of ME-based polymer gels was assessed against standard (ATCC 10231) and clinical strains (including the fluconazole-resistant strain) of *Candida albicans* using the agar diffusion method. The inhibition zone was measured to determine the activity of VRC delivered via the topical systems against *Candida albicans*. All of the fungal strains were cultured on Malt extract agar (Merck KGaA, Darmstadt, Germany) under aerobic conditions for 24 h and at 36 ± 1 °C. Following incubation, the cultures were resuspended in a 0.85% NaCl solution. The turbidity of the suspension was measured using a Grant-Bio DEN-1 Benchtop Densitometer (Riga, Latvia) and adjusted to a value of 0.7 McFarland units (corresponding to approximately 2.0 × 10^6^ CFU/1 mL for *C. albicans*; CFUs—Colony Forming Units). Then, a sterile cotton swab was dipped into the suspension and evenly spread over the entire surface of the agar plate by swabbing in three directions. Next, the wells of 6 mm in diameter were punched aseptically with a sterile pipette tip, and 0.1 mL of the gel was applied manually to the well. The study involved four different gels—B_2_, B_3_, B_4_, and placebo (P) (Table 2). The plates were incubated at 36 ± 1 °C. The inhibition zones were measured at 24 and 48 h using a ruler with an accuracy of 1 mm to evaluate fungal growth inhibition. All assays were determined in triplicate to evaluate the mean and standard deviation (SD).

#### 2.2.16. Statistical Analysis

Statistical analysis was performed using Statistica software, version 13.3.721.1 (TIBCO Software Inc., Palo Alto, CA, USA). The normality of data distribution was assessed using the Shapiro–Wilk test. For variables meeting the assumption of normality, the Brown–Forsythe test was applied to evaluate the homogeneity of variances, followed by analysis of variance (ANOVA) with post hoc tests: Scheffé’s test and Tukey’s HSD test for unequal sample sizes. For non-normally distributed data, nonparametric tests were used, including the Kruskal–Wallis ANOVA and the Mann–Whitney U test. A significance level of *p* < 0.05 was adopted for all analyses.

All graphs were generated using OriginPro, Version 2025 (OriginLab Corporation, Northampton, MA, USA), unless otherwise indicated.

## 3. Results

### 3.1. Microemulsion Preparation and Conductivity Studies

Screening studies were conducted to identify MEs with the greatest potential to form stable, transparent mixtures of the oil phase, surfactant, co-surfactant, and water. Based on these preliminary evaluations, Gibbs phase diagrams were constructed to visualize the ME regions. These diagrams allowed for the selection of formulations exhibiting the largest clear phase domains, indicative of optimal ME formation. From the systems evaluated, five formulations, ME A_9.1_, A_13.1_, A_16.1_, A_19.1_, and A_23.1_, were selected for further investigation. All of these systems shared a fixed surfactant-to-co-surfactant ratio (S:CoS) of 1:1 (*w*/*w*), using Brij^®^ O5-SS-(RB) as the surfactant and Transcutol^®^ as the co-surfactant. The selected systems differed in their oil phase composition, which included triacetin, isopropyl palmitate, ethyl oleate, oleic acid, and isopropyl myristate, respectively.

**Figure 2:**
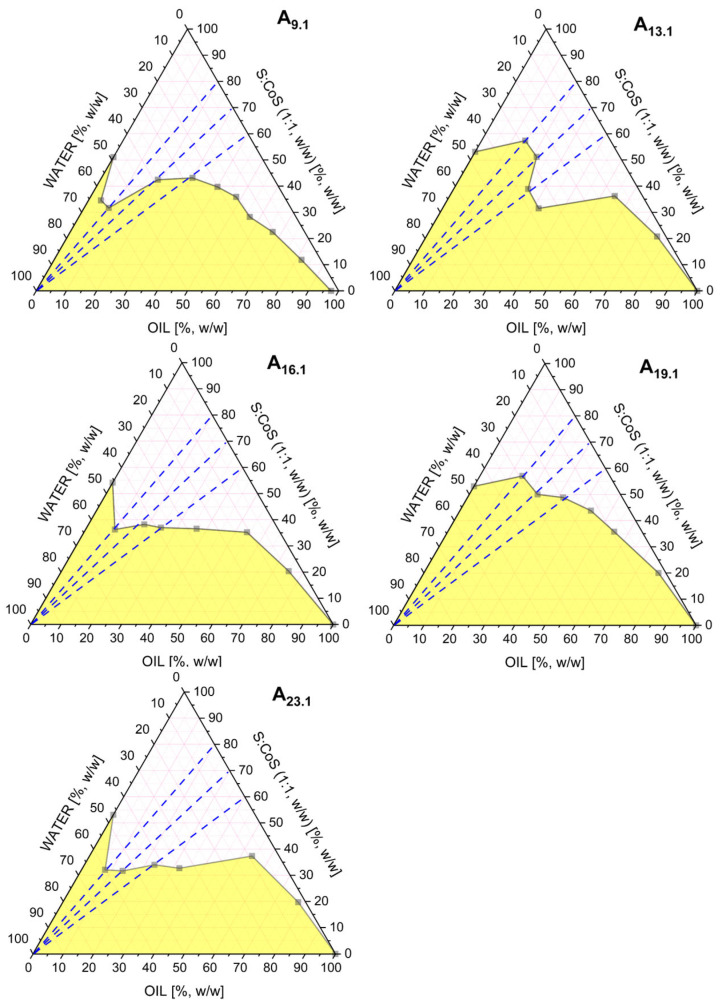
Pseudoternary phase diagrams obtained for the MEs A_9.1_, A_13.1_, A_16.1_, A_19.1_, and A_23.1_, differing in the type of oil phase used (triacetin, isopropyl palmitate, ethyl oleate, oleic acid, and isopropyl myristate, respectively). All systems contained a fixed surfactant-to-co-surfactant mixture (Brij^®^ O5-SS-(RB)/Transcutol^®^, 1:1 *w*/*w*). The white region represents the ME area, while the yellow region corresponds to multiphase or turbid systems. The blue dashed line indicates the dilution pathway used in the conductivity study.

The corresponding phase diagrams for these selected mixtures are presented in Figure 2.

The MEs A_9.1_, A_13.1_, A_16.1_, A_19.1_, and A_23.1_ were further evaluated by electrical conductivity measurements. These studies were performed along three different oil-to-S:CoS weight ratios, 4:6, 3:7, and 2:8, following the dashed blue dilution lines indicated in Figure 2.

The conductivity profiles, presented in Figures S1–S5 (Supplementary Materials), show the changes in electrical conductivity (μS·cm^−1^) as a function of water phase content (%, *w*/*w*). In all tested systems, a phase transition from a water-in-oil (w/o) to a bicontinuous ME structure was observed with increasing water content. However, only in the A_16.1_ system, additional phase transitions from bicontinuous to oil-in-water (o/w) MEs were identified at oil-to-S:CoS ratios of 4:6 and 3:7. This o/w transition was not observed in the other systems.

Based on the conductivity results, five ME formulations were selected for further investigation. All systems differed only in the type of oil phase. For the next stage of formulation, the oil-to-S:CoS weight ratio of 3:7 was chosen, and the water content was fixed at 23% (*w*/*w*).

### 3.2. Solubility Studies

Solubility studies were conducted for selected components of the MEs, including five oil phases (triacetin, oleic acid, ethyl oleate, isopropyl palmitate, and isopropyl myristate), three surfactants (Tween^®^ 80, Ethoxylated Castor Oil—Etocas^TM^ 35, and Brij^®^ O5-SS-(RB)), and one co-surfactant (Transcutol^®^). The choice of these media was based on the compositions of the selected MEs and their potential use in further formulation development.

The solubility of VRC in each medium was determined at two temperatures: 25 °C and 32 °C. The results are presented in Table 3.

Importantly, the applied experimental method did not allow for a reliable determination of VRC solubility in Etocas^TM^ 35 and Tween^®^ 80. Despite multiple attempts, the measurements could not be completed due to the high viscosity of both solvents and the low melting point of Etocas^TM^ 35. The solubility test was performed in vials enabling mixing from the top, bottom, or both simultaneously. However, in all approaches, the viscosity of these media was too high to ensure proper dispersion of VRC. As a result, undissolved VRC sediment remained at the bottom of the vials after the experiments. Thus, alternative solubility testing methods should be considered in future studies for these two surfactants.

### 3.3. In Vitro Release Studies of Voriconazole from Microemulsions

**Figure 3:**
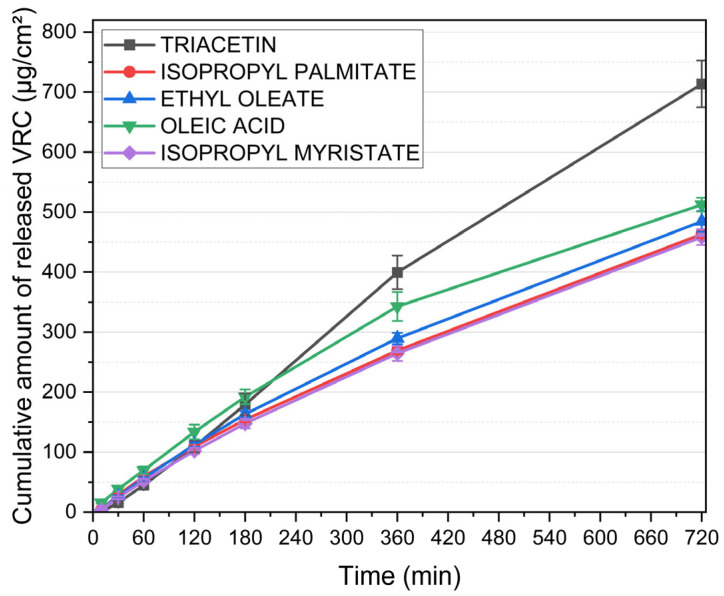
Cumulative release of voriconazole from microemulsions containing different oil phases. Water content (23%) and S:CoS mixture (Brij^®^ O5-SS-(RB)/Transcutol^®^, 1:1 *w*/*w*) were constant across all formulations; the oil-to-S:CoS ratio was maintained at 3:7 (*w*/*w*). Data are presented as mean ± standard deviation (n = 6).

Figure 3 illustrates the cumulative release profiles of VRC from MEs formulated with various oil phases. All systems contained 23% water and a constant S:CoS mixture composed of Brij^®^ O5-SS-(RB) and Transcutol^®^ (1:1, *w*/*w*), with an oil-to-S:CoS ratio of 3:7 (*w*/*w*), and 1% (*w*/*w*) VRC. Despite identical aqueous and surfactant compositions, significant differences in VRC release were observed depending on the oil phase used.

The formulation containing triacetin demonstrated the highest drug release, reaching over 713 ± 39 µg/cm^2^ within 720 min. This was markedly higher than the release achieved by systems based on other oils. MEs incorporating oleic acid and ethyl oleate exhibited intermediate release rates, while the lowest cumulative amounts of VRC were released from formulations with isopropyl palmitate and isopropyl myristate.

Based on these results, subsequent formulation efforts were focused on systems incorporating triacetin as the oil phase, due to its superior performance in promoting voriconazole release compared to the other tested oils.

### 3.4. Optimization of Microemulsion Composition and 2nd Conductivity Studies

**Figure 4:**
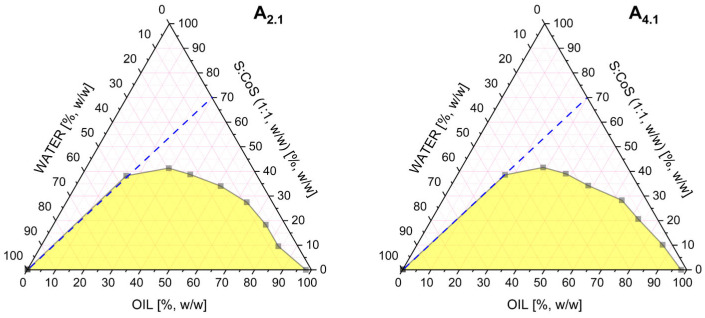
Pseudoternary phase diagrams obtained for the microemulsions A_2.1_ and A_4.1._ differing in the type of surfactant used. All systems contained a fixed surfactant-to-co-surfactant mixture (1:1 *w*/*w*). The white region represents the microemulsion area, while the yellow region corresponds to multiphase or turbid systems. The blue dashed line indicates the dilution pathway used in the conductivity study.

**Figure 5:**
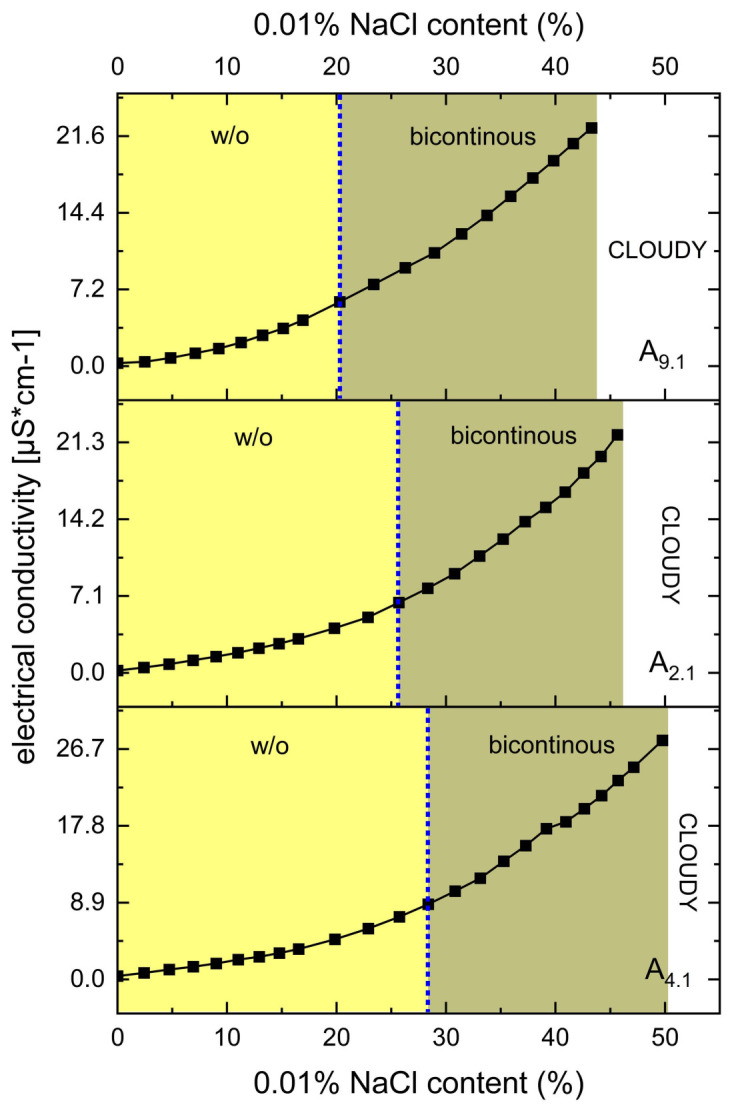
Electrical conductivity [μS·cm^−1^] as a function of 0.01% NaCl phase content [%, *w*/*w*] for systems A_9.1_, A_2.1_, and A_4.1_ at oil-to-S:CoS ratios of 3:7. The blue dotted lines indicate the approximate structural transition points. All systems contain 1% (*w*/*w*) of VRC.

Based on the release studies presented in Section 3.3, formulation A_9.1_, containing triacetin as the oil phase, was selected for further investigation. Two additional MEs were subsequently identified for comparative analysis. These formulations maintained the same oil phase (triacetin) and co-surfactant (Transcutol^®^), but differed in the surfactant component: A_2.1_ employed Tween^®^ 80, while A_4.1_ utilized Etocas^TM^ 35. The respective Gibbs phase diagrams for these two systems are shown in Figure 4. All three formulations were subjected to conductometric titration to assess the structural transitions occurring upon aqueous phase addition. These measurements were performed using a previously established oil-to-S:CoS (1:1 *w*/*w*) mass ratio of 3:7. The resulting plots of electrical conductivity (μS·cm^−1^) as a function of water phase content (%, *w*/*w*) for systems are presented in Figure 5.

The results, presented in Figure 5, clearly indicate that none of the systems (A_9.1_, A_2.1_, and A_4.1_) undergo a transition from the bicontinuous structure to an o/w ME. This is evidenced by the continuous and gradual increase in electrical conductivity with increasing water content, without a sharp inflection point that would typically signify a structural transition to an o/w system.

Despite the absence of such a transition, the conductometric data were instrumental in selecting the appropriate water content for further formulation development. Based on the conductivity profiles, a water content of 38% was established as optimal. At this concentration, stable MEs were formed within the bicontinuous region, offering a suitable environment for subsequent studies and potential applications.

### 3.5. Ex Vivo Skin Permeation and Skin Deposition Studies of Voriconazole (From ME)

**Figure 6:**
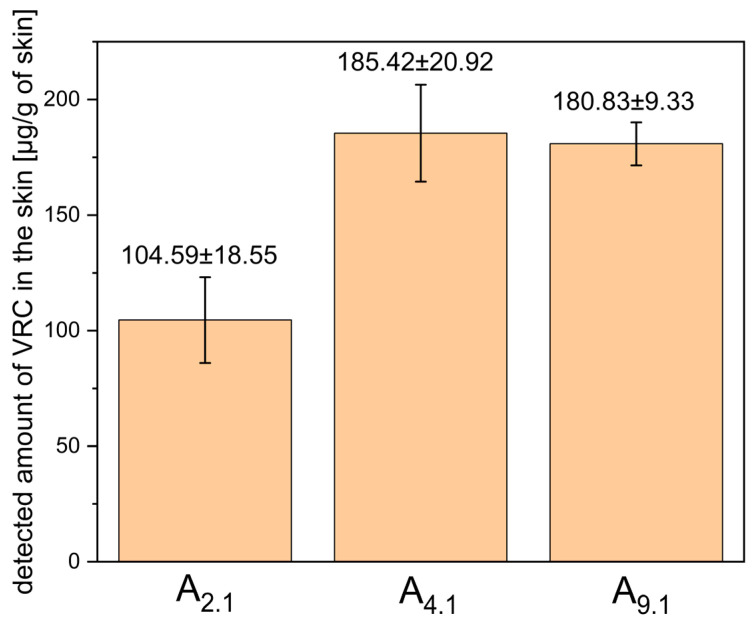
Detected amount of VRC in human skin (epidermis + dermis) after 24-h ex vivo permeation study using microemulsions A_2.1_, A_4.1_, and A_9.1_. Results are expressed as mean ± SD (n = 3).

MEs A_9.1_, A_2.1_, and A_4.1_ were subjected to ex vivo permeation studies using full-thickness human skin to evaluate the transdermal delivery and skin deposition of VRC. After 24 h, VRC was detected in the acceptor fluid only for system A_4.1_, indicating its ability to facilitate transdermal permeation. In contrast, for systems A_2.1_ and A_9.1_, the amount of VRC that permeated the skin remained below the detection limit of the analytical method. The quantitative data on VRC deposition in the skin, including both the epidermis and dermis layers, are presented in Figure 6.

Among the tested systems, A_4.1_ showed the highest level of VRC accumulation in the skin, although the difference was not statistically significant compared to system A_9.1_ (*p* = 0.349). Based on these results, system A_4.1_ was selected for further studies due to its superior microemulsion stability, higher capacity for water phase incorporation, and its ability to enable transdermal delivery of VRC.

### 3.6. Physicochemical Characterization of Selected MEs

The selected ME system A_4.1_ was subjected to physicochemical characterization, including the determination of pH, polydispersity index (PDI), particle size, viscosity, and refractive index (RI). Additionally, a 2% menthol additive was incorporated into the formulation, given the well-documented role of this terpenol both as a permeation enhancer and an antifungal agent. The obtained systems were transparent, and no phase separation was observed upon the addition of the drug and menthol. The compositions of the systems are presented in Table 4, while the results of the conducted analyses are summarized in Table 5.

### 3.7. Physicochemical and Functional Characterization of Microemulsion-Based Gels

Based on the optimized MEs, ME-based hydrogels (ME-hydrogels) were subsequently developed to obtain formulations with enhanced applicability for topical administration. These systems were subjected to comprehensive characterization, including the assessment of pH, drug content, rheological behavior, and spreadability. The compositions of ME-hydrogels with their pH values are presented in Table 2.

The results of the drug content analysis are presented in Table 6.

#### 3.7.1. Rheological Studies

**Figure 7:**
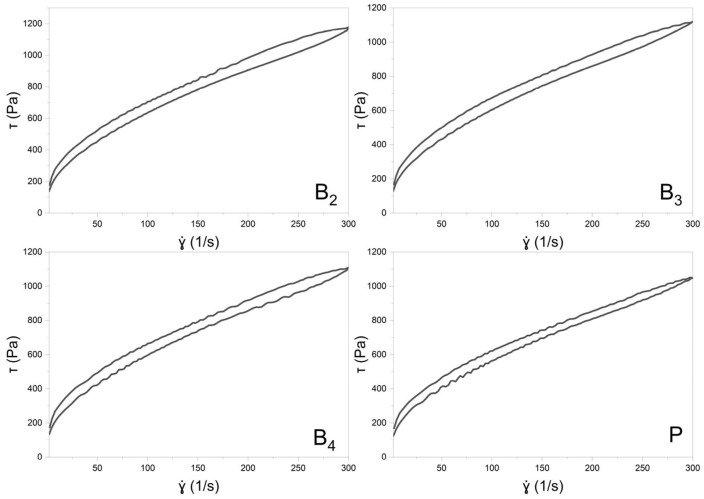
The flow curves of formulation B_2_, B_3_, B_4_, and P (PLACEBO) with a hysteresis loop.

**Figure 8:**
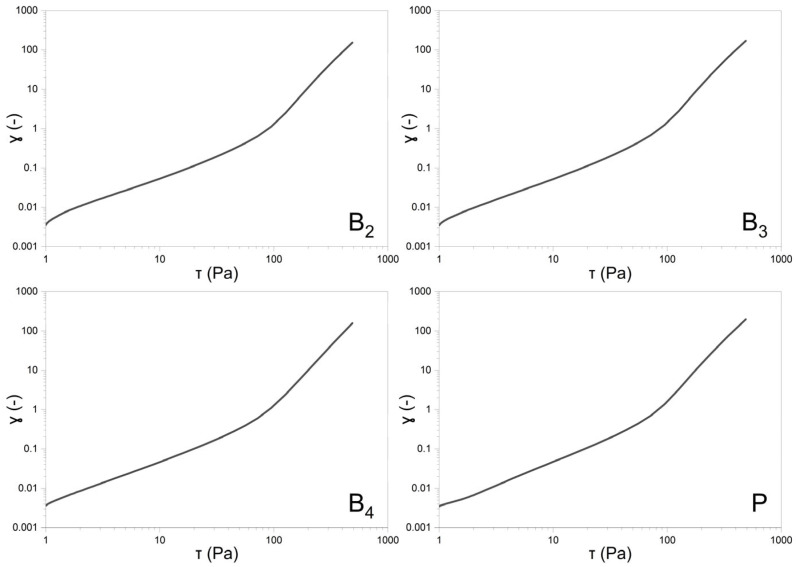
Double-logarithmic plots of sample deformation vs. shear for formulations B_2_, B_3_, B_4_, and P (PLACEBO).

Rheological evaluations included both steady shear and oscillatory measurements to characterize the viscoelastic behavior of the formulations. The flow curves obtained under controlled shear rate (CR) and controlled stress (CS) conditions are shown in Figure 7 and Figure 8, respectively. Data from the CR experiments were modeled using the Herschel–Bulkley equation [20]:

$$\tau =\;\tau_{0}+K{\dot{\gamma }}^{n}$$

where τ represents the shear stress, $\tau_{0}$ is the yield stress, K denotes the consistency index, $\dot{\gamma }$ is the shear rate, and *n* is the flow behavior index. For the CS measurements, yield points were determined by identifying the intersection of two tangents fitted to the near-linear segments of the stress–strain curve. All the calculated parameters are summarized in Table 7.

#### 3.7.2. Spreadability Test

**Figure 9:**
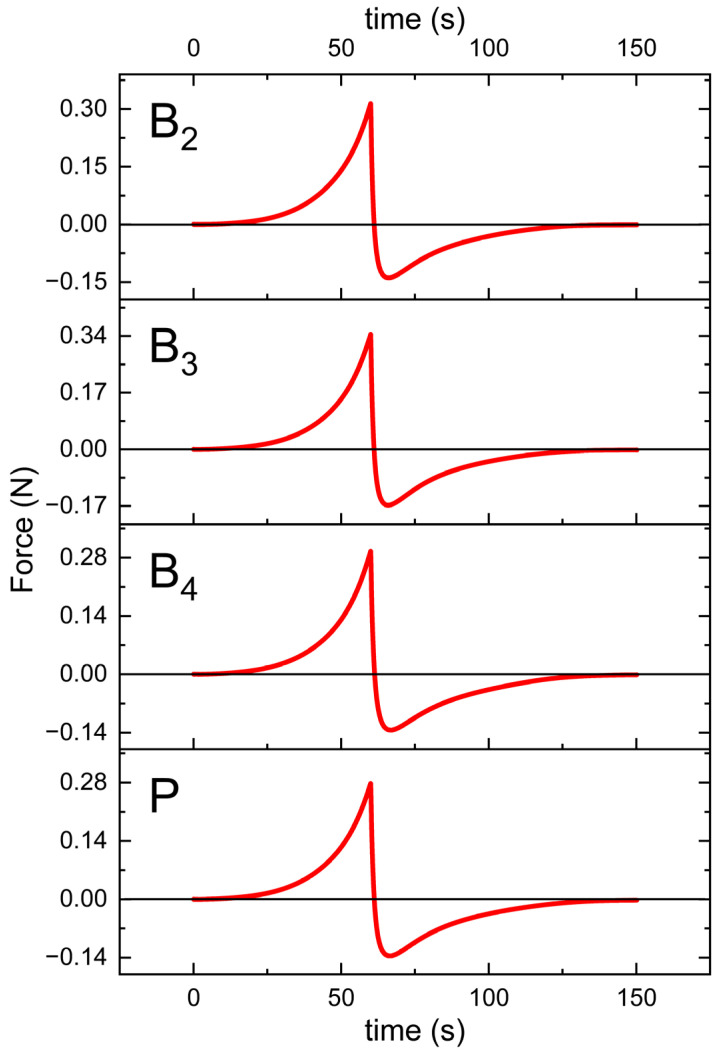
Texture profiles of the formulations B_2_, B_3_, B_4_, and P (placebo) plotted as a force against time.

The texture profiles obtained during the spreadability test are presented in Figure 9, whereas Table 8 presents the values of the test parameters that were determined.

### 3.8. Ex Vivo Skin Permeation and Skin Deposition Studies of Voriconazole (From ME-Hydrogels)

**Figure 10:**
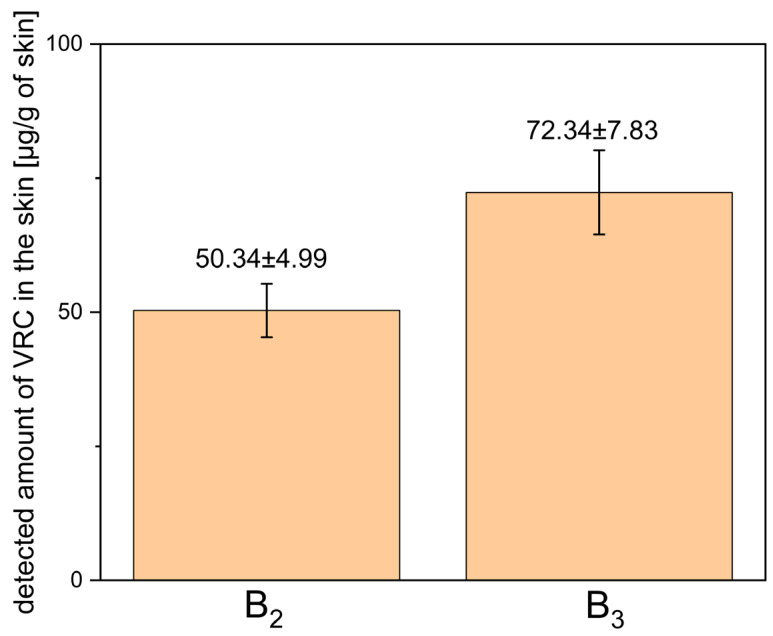
Detected amount of voriconazole in human skin (epidermis + dermis) after 24-h ex vivo permeation study using ME-hydrogels B_2_ and B_3_. Results are expressed as mean ± SD (n = 3).

ME-hydrogels B_2_ and B_3_ were subjected to *ex vivo* permeation studies using full-thickness human skin to evaluate the transdermal delivery and skin deposition of VRC. After 24 h, the concentration of VRC in the acceptor fluid was below the level of detection. The quantitative data on VRC deposition in the skin, including both the epidermis and dermis layers, are presented in Figure 10.

### 3.9. Antifungal Properties

**Figure 11:**
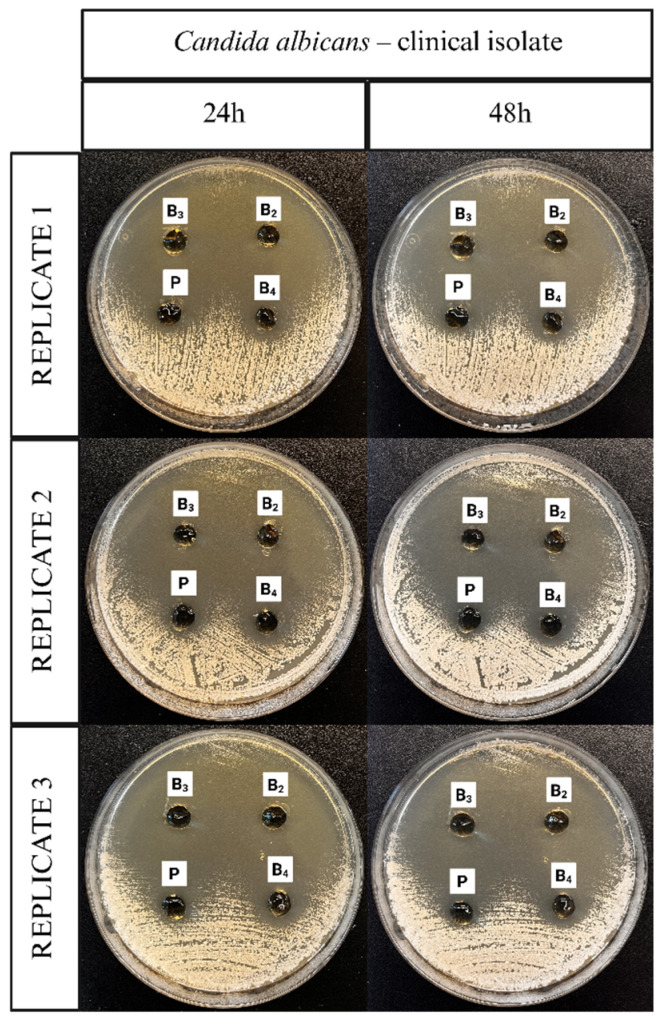
Representative images documenting the application of ME-hydrogels on agar plates inoculated with *C. albicans* clinical isolate strain.

**Figure 12:**
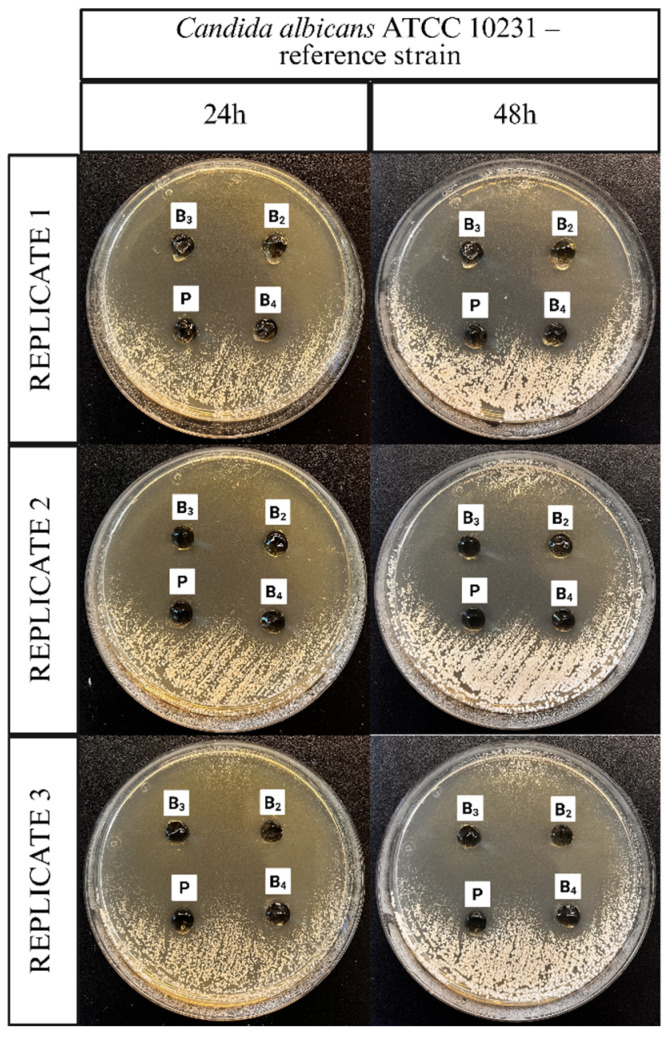
Representative images documenting the application of ME-hydrogels on agar plates inoculated with *C. albicans* ATCC 10231 reference strain.

**Figure 13:**
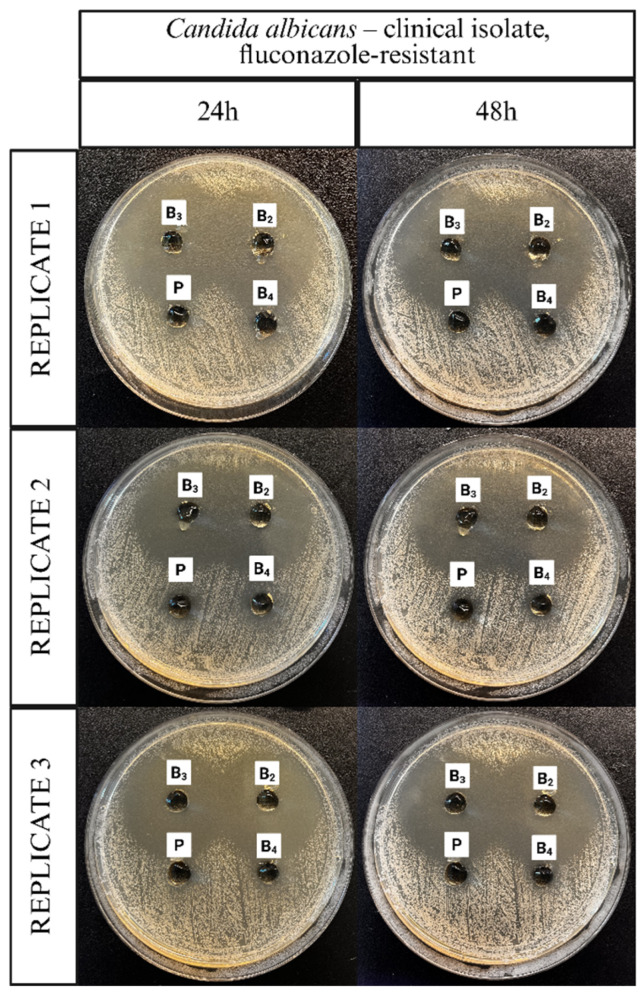
Representative images documenting the application of ME-hydrogels on agar plates inoculated with *Candida albicans* clinical isolate, fluconazole-resistant strain.

The antifungal activity of the ME-hydrogels was evaluated using agar diffusion assays against different *C. albicans* strains. Representative images illustrating the application of the ME-hydrogels on agar plates inoculated with a clinical isolate of *C. albicans*, the *C. albicans* ATCC 10231 reference strain, and a fluconazole-resistant clinical isolate of *C. albicans* are presented in Figure 11, Figure 12 and Figure 13.

**Figure 14:**
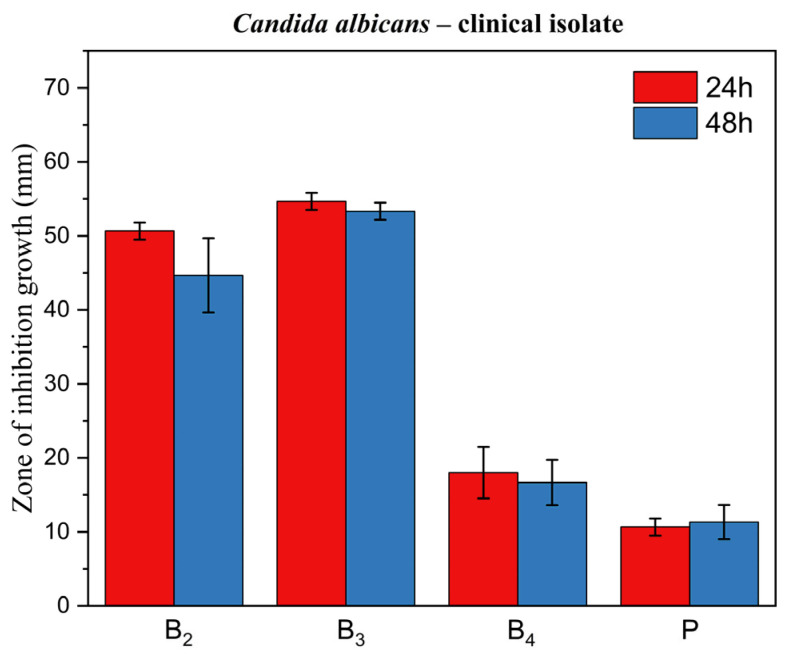
Bar chart: zone of inhibition of growth for ME-based polymer gels with VRC against *Candida albicans* clinical strain after 24 and 48 h of incubation.

**Figure 15:**
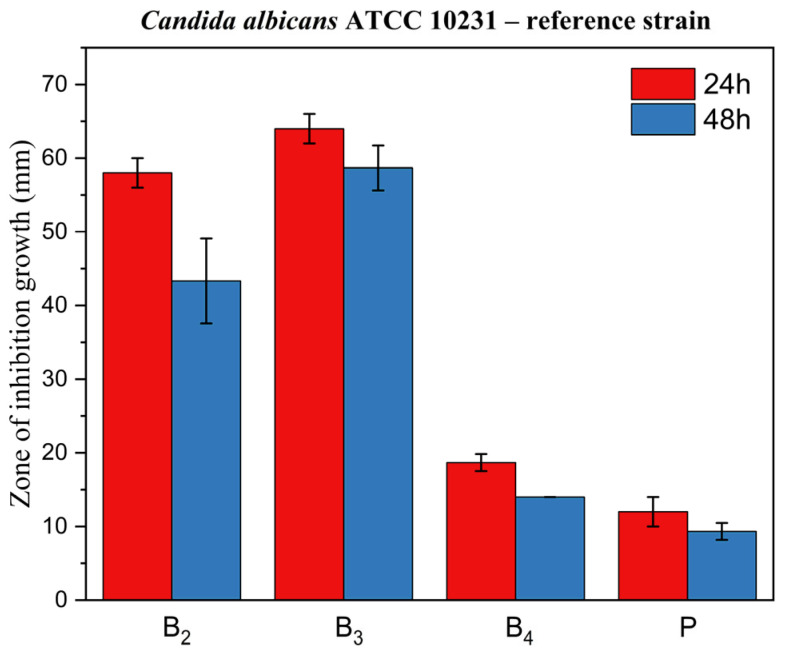
Bar chart: zone of inhibition of growth for ME-based polymer gels with VRC against *C. albicans* ATCC 10231 reference strain after 24 and 48 h of incubation.

**Figure 16:**
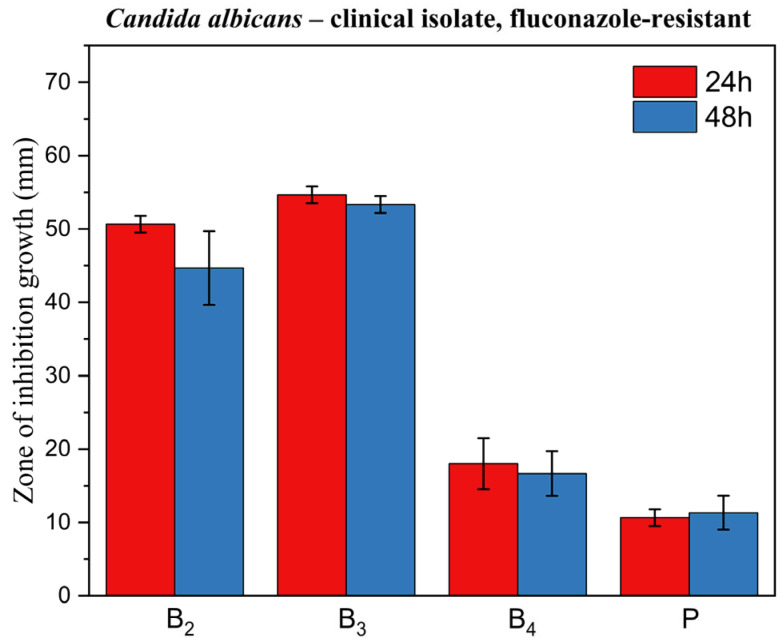
Bar chart: zone of inhibition of growth for ME-based polymer gels with VRC against *Candida albicans* clinical fluconazole-resistant strain after 24 and 48 h of incubation.

Figure 14, Figure 15 and Figure 16 display corresponding bar charts quantifying the zones of inhibition for the ME-based polymer gels containing VRC against the same strains after 24 and 48 h of incubation: clinical *C. albicans* strain, ATCC 10231 reference strain, and fluconazole-resistant clinical isolate. These results demonstrate the antifungal efficacy of the formulations over time across different *C. albicans* variants.

## 4. Discussion

Screening of formulation components led to the evaluation of 46 MEs. Based on the screening results and initial conductometric analyses, a fixed S:CoS ratio (1:1, *w*/*w*) was selected together with five oil phases, triacetin, isopropyl palmitate, ethyl oleate, oleic acid, and isopropyl myristate, which were formulated with 1% VRC and subjected to in vitro release testing. Transcutol^®^ was chosen as a co-surfactant as it is a pharmaceutically accepted penetration enhancer with a well-documented safety profile [21]. Its superior solubilization capacity for both hydrophilic and lipophilic actives, combined with miscibility across a broad range of excipients, facilitates the formation of stable MEs and supports enhanced drug partitioning into the skin [21,22]. Mechanistically, Transcutol^®^ can readily penetrate the stratum corneum, interact with intercellular water, and reversibly increase skin permeability without compromising structural integrity [21]. Toxicological evaluations indicate that high-purity Transcutol^®^ is well tolerated across species [23]. Its non-irritant or only slightly irritating nature in dermal and ocular tests further supports suitability for topical delivery systems [23].

Given its strong enhancing effect on drug flux in MEs, broad compatibility, and robust nonclinical safety record, Transcutol^®^ represents an optimal choice as a co-surfactant for transdermal and topical drug delivery formulations.

In vitro release experiments demonstrated that the triacetin-based formulation provided a significantly higher VRC release compared to the other four oil phases (*p* < 0.05). According to the mathematical model proposed by Grassi et al., the main determinants of drug release from MEs include drug solubility in the oil phase, the kinetic transfer constants between phases (k_ow_ and k_wo_), and the interfacial area (A), in addition to Fick’s law of diffusion [24]. The model also predicts that a low ratio of drug solubility in the aqueous phase to that in the oil phase (RS) tends to decrease the release rate, since the drug strongly partitions into the oil phase [24].

When applying this model to our findings, triacetin exhibits the highest VRC solubility among the oils tested, providing a large local reservoir of drug within each droplet (high C_0_). While a high C_0_ alone does not necessarily guarantee faster release, in this case, it is accompanied by a set of favorable physical properties. Triacetin has low viscosity and the smallest molecular weight of the oils tested, which together promote rapid intra-droplet diffusion and likely increase k_ow_ by reducing mass-transfer resistance. Additionally, triacetin is the only oil phase among those investigated that is miscible with water (~60 g/L) and exhibits a very low log P (~0.044). This partial water solubility may lead to droplets with diffuse or “blurred” oil–water interfaces, effectively increasing the interfacial area available for mass transfer.

Droplet size measurements further support this interpretation: triacetin-based microemulsions displayed extremely small droplet diameters but relatively high polydispersity indices (PDI). The elevated PDI may result from secondary scattering effects due to the high droplet concentration, but also reflects a system with a very large total interfacial area. Taken together, the combination of high C_0_, potentially elevated k_ow_ due to low viscosity and small molecular size, and a large effective interfacial area appears to compensate for the theoretically unfavorable low RS, enabling rapid drug transfer into the aqueous phase. Although this mechanistic explanation requires further verification through targeted experiments, the superior permeation observed in both in vitro studies justified the selection of triacetin as the oil phase for subsequent development work. Furthermore, literature reports indicate that triacetin has been widely employed as an oil phase in the development of topical MEs, where it not only enhances drug solubilization but also contributes to favorable droplet characteristics, stability, and skin delivery performance, making it a versatile excipient for dermal and transdermal applications [25].

Following the selection of the oil phase and co-surfactant, the next stage of the study assessed the influence of different surfactants on the transdermal permeation of VRC through full-thickness human skin and its deposition within the skin layers. Three surfactants with varying hydrophilic–lipophilic balance (HLB) values were investigated: Brij^®^ O5-SS-(RB) (HLB 9.10), Etocas^TM^ 35 (HLB 12.7), and Tween^®^ 80 (HLB 15.0). The combination of the selected oil phase and Transcutol^®^ as co-surfactant enabled the incorporation of a relatively high proportion of the aqueous phase into the microemulsions. Notably, the Etocas^TM^ 35-based formulation allowed incorporation of up to 50% aqueous phase at 32 °C, whereas the Brij^®^ O5-based system exhibited the lowest capacity for water loading. Conductometric titration did not indicate a transition of the systems to an o/w microemulsion type. Nevertheless, conductometric analysis identified 38% aqueous phase content as optimal to maintain stability across all three systems.

Non-ionic surfactants differ markedly in HLB, ethoxylation, and chain length, and these parameters strongly influence microemulsion penetration enhancement. In general, moderately lipophilic surfactants (shorter ethylene oxide (EO) chains, HLB ≈ 7–9) maximize flux by partitioning into stratum corneum lipids, whereas very hydrophilic surfactants (long EO tails, high HLB) tend to stay in the aqueous phase and give lower flux [26]. For example, Park et al. showed that polyoxyethylene (2–5) alkyl ethers (HLB ≈ 7–9, C16–C18 tails) produced the greatest ibuprofen flux [27]. Consistent with this, Kim et al. found that polyoxyethylene-2-oleyl ether (a Brij-type surfactant with only 2 EO units) roughly doubled bupivacaine permeation relative to controls [28]. In mixed systems, the overall HLB still governs permeation: Vu et al. observed that a Tween-80/Brij^®^-30 blend (intermediate HLB) gave higher genistein flux than Tween^®^ 80 alone, whereas increasing the blend HLB steadily decreased flux [29]. Mechanistically, lipophilic surfactants (e.g., Brij^®^ O5, an oleyl ether) insert into and fluidize stratum corneum lipids more efficiently than highly ethoxylated ones (e.g., Etocas^TM^ 35, PEG-35 castor oil, or Tween^®^ 80), which tend to solubilize the drug in the vehicle and slow release [26,29]. However, beyond surfactant selection, the proportion of aqueous phase loaded into the microemulsion is also a critical determinant of performance, as higher water content often facilitates higher drug release from the formulation by promoting drug partitioning into the skin [30]. Thus, selecting nonionic surfactants with the appropriate chain length, degree of ethoxylation, and optimizing aqueous phase content are key factors for maximizing skin permeation efficiency.

Our results demonstrated that transdermal delivery of VRC was achieved only with the ME containing Etocas^TM^ 35 as the surfactant, whereas systems formulated with Tween^®^ 80 or Brij^®^ O5 showed no detectable VRC in the receptor phase, with concentrations remaining below the analytical method’s limit of detection. In terms of skin deposition (epidermis + dermis), both the Etocas^TM^ 35- and Brij O5-based systems exhibited comparable VRC levels (*p* > 0.05) of approximately 180 µg/g of skin. These findings are consistent with the mechanistic considerations described above, whereby surfactants with lower HLB values tend to promote greater skin penetration. Nevertheless, given that the Etocas^TM^ 35 system demonstrated superior stability, higher aqueous phase loading capacity (up to 50% at 32 °C), and measurable transdermal delivery, formulation 4.1 (Etocas^TM^ 35-based) was selected as the final ME. This formulation was subsequently subjected to physicochemical characterization and gelation for further evaluation. When interpreting these findings, it is important to consider not only the general principles related to HLB but also the molecular features of the individual surfactants. The absence of detectable transdermal delivery from the Tween^®^ 80 system is consistent with its high HLB and strong hydrophilicity, which favor drug retention in the aqueous phase rather than partitioning into the skin, and also aligns with its lowest observed VRC deposition. However, the superior performance of Etocas™ 35 compared with Brij^®^ O5 cannot be fully explained by HLB values alone. Etocas™ 35, a PEG-35 castor oil derivative, possesses a large hydrophilic head group with 35 EO units and a high molecular weight, which enables the incorporation of high water volumes and the formation of smaller, more uniform oil droplets. Literature evidence confirms that Etocas™ 35 is particularly effective in generating fine dispersions, whereas relatively lipophilic surfactants such as Brij^®^ O5 (only 5 EO units) tend to form larger, oil-rich droplets [31]. Smaller droplet size increases the interfacial surface area available for drug release and enhances drug–skin contact, providing a mechanistic explanation for the enhanced permeation observed with Etocas™ 35.

In addition, structural differences in the hydrocarbon tails are likely to contribute. Etocas™ 35 is composed mainly of ricinoleate esters, whereas Tween^®^ 80 and Brij^®^ O5 are based on straight oleyl chains. The hydroxyl group on ricinoleic acid imparts additional polarity and hydrogen-bonding capacity, which may promote stronger interactions with SC lipids and proteins [26]. By inserting its bulky glyceride/PEG structure into the SC, Etocas™ 35 is more likely to fluidize and partially solubilize the lipid lamellae than the simpler oleyl-based surfactants. Thus, Etocas™ 35 appears to act through a combination of mechanisms: producing finer emulsions with higher water loading (and therefore greater drug availability at the skin interface), while simultaneously exerting stronger disruptive effects on the SC lipid barrier due to the ricinoleate tail. In contrast, Tween^®^ 80 and Brij^®^ O5 disrupt SC bilayers primarily by inducing packing defects, which are comparatively weaker in terms of barrier perturbation.

Taken together, the high degree of ethoxylation and unique ricinoleate-based tail structure of Etocas™ 35 provide synergistic advantages in both droplet formation and SC lipid disruption, possibly explaining why this surfactant outperformed Tween^®^ 80 and Brij^®^ O5 in enabling transdermal delivery of voriconazole.

Given the well-documented properties of menthol both as a permeation enhancer and an antifungal agent, an additional formulation was developed containing 2% menthol (*w*/*w*). Menthol has been shown to enhance percutaneous drug absorption primarily by disrupting and fluidizing the lipid bilayers of the stratum corneum, thereby increasing skin permeability [32]. Moreover, menthol exhibits intrinsic antifungal activity, with demonstrated efficacy against dermatophytes and *Candida* species, which has been attributed to membrane disruption and leakage of intracellular components [33]. In view of these properties, two additional ME variants were prepared: one containing both menthol (2%) and VRC, and another containing menthol alone.

The measured pH values were comparable across all investigated MEs, with the exception of the placebo formulation, which exhibited a slightly higher pH. All microemulsions displayed Newtonian flow behavior, characterized by constant viscosity across the entire shear rate range, as typically observed for such systems [34]. The viscosity values for all samples were approximately 41 mPa·s. DLS analysis indicated particle sizes within the nanometric range, approximately 2.8–3 nm. This size distribution may suggest localization of the drug molecules within the interfacial layer. However, it should be noted that MEs are concentrated systems prone to multiple scattering effects and cannot be diluted without inducing significant structural changes. As highlighted by other authors, without the application of appropriate corrections, DLS results for such systems should be interpreted with caution [35]. Importantly, the exceptionally small droplet size may enhance transdermal drug delivery by increasing the surface area for drug release, improving close contact with the stratum corneum, and potentially facilitating diffusion through the skin barrier.

The MEs were successfully gelled using Carbopol^®^ and TIPA (Table 2). The pH values of the resulting gels were in the range of 5.35–5.49, so within the range of 4–6, which is generally considered suitable for dermal application [36]. Loading with both VRC and menthol decreases the pH of the system. The gels were subjected to rheological and texture profile analysis. Rheological evaluation revealed a non-Newtonian, shear-thinning flow behavior typical of Carbopol^®^-based semisolid gels [37]. Under increasing shear stress, hydrogen bonds and other weak interactions between polymer chains forming the three-dimensional gel network are progressively disrupted. The gradual alignment of polymer chains in the flow direction results in a decrease in viscosity with increasing shear rate. Such behavior is advantageous for dermal products intended for skin spreading, as the viscosity drop upon rubbing facilitates a smoother and more comfortable application [38].

The shear-thinning nature of all analyzed gels is reflected by the flow behavior index *n* calculated according to the Herschel–Bulkley model, which remained below 1 (Table 7). While the n values did not differ significantly among the formulations (*p* = 0.1574), the consistency index (K) was lower for the placebo gel. The addition of VRC and menthol appeared to increase K, with the lowest value observed for the placebo, intermediate values for gels containing either menthol or VRC alone, and the highest for the formulation containing both. Regarding yield stress determined in controlled stress (CS) mode, the gel containing VRC alone exhibited significantly higher values compared to both the B_3_ gel and the placebo gel (*p* < 0.05), suggesting a potential strengthening effect of VRC on the gel network structure. In terms of τ_0_, although one-way ANOVA indicated statistically significant differences between groups, subsequent post hoc analysis did not reveal significant differences between any specific pair of gels.

Thixotropy, expressed as the relative hysteresis area (Kd), was low for all investigated gels (Table 7). Low thixotropy values are generally considered favorable for topical preparations, as they indicate rapid structural recovery after shear, ensuring product stability during storage and maintaining consistent performance upon application. Conversely, high thixotropy may prolong the recovery time, which can be advantageous in some contexts (e.g., injectable depot systems) but less desirable for dermal gels, where prompt recovery of viscosity after spreading helps maintain the formulation at the site of application [39].

In spreadability tests, the B_3_ gel required the most force and energy to spread, while the plain placebo gel required the least (Table 8). One-way ANOVA indicated statistically significant differences between groups, but subsequent post hoc analysis did not reveal significant differences between any specific pair of gels. Nevertheless, these trends reflect the rheological profiles (Table 7); B_3_ had the highest consistency index, whereas the placebo had the lowest consistency. This is consistent with the established understanding that higher viscosity or yield stress typically makes a semisolid harder to spread [40]. In our gels, the formulations with greater rheological strength (higher K) also required greater firmness and spread energy in the texture analysis, reflecting this general correlation between rheology and spreadability. These findings are in line with the concept that texture parameters such as firmness (hardness) and adhesiveness tend to rise as the internal structure or polymer network becomes stronger. Texture profile analysis (TPA) in this context provides quantitative measures of semisolid consistency. By compressing the gel and recording force–time curves, TPA yields parameters such as firmness (the peak force) and adhesiveness (the work required to withdraw the probe from the sample), which are directly relevant to how a product feels and behaves on the skin. As expected, formulations containing additives were firmer than the placebo. Adding VRC (B_2_ vs. P) led to a modest increase in firmness, suggesting the drug slightly reinforced the gel network. Adding menthol (B_3_ vs. B_2_, and B_4_ vs. P) produced further increases in this parameter. Interestingly, other researchers have observed the opposite effect of menthol in different systems: Otto et al. found that menthol decreased the yield stress, hardness, and adhesiveness of a ketoprofen ME gel [18]. The most likely explanation for the observed differences in our study is the distinct preparation method used for menthol-containing gels, which could have significantly influenced the mechanical properties of the formulations (see Section 2.2.10. Additionally, it should be noted that the way menthol interacts with the system and its impact on gel properties also depends on formulation specifics, such as polymer type, oil phase, and other components. Thus, menthol (which is solid at room temperature and somewhat crystalline) may act as a reinforcing filler in the Carbopol^®^ matrix or alter water–polymer interactions, thus increasing gel cohesion. In sum, B_3_ showed the greatest firmness, followed by B_2_ and B_4_, with the placebo being the lowest. These differences, however, were not statistically significant (*p* = 0.1079).

It is important to emphasize that these texture measurements were made in vitro, in a container, without any skin. Real skin presents friction, compliance, micro-texture, and temperature effects that are not captured here, so in vivo spreadability may differ. Nonetheless, the instrumental results provide useful relative information about product feel.

The microbiology results confirm the antifungal efficacy of the developed ME polymer gels containing VRC against various *Candida albicans* strains, including both reference and clinical isolates, as well as a fluconazole-resistant strain. The observed inhibition zones demonstrate that the incorporation of VRC into the ME-based gel results in potent antifungal activity, consistent with literature reports on the effectiveness of VRC in topical delivery systems.

Al-Suwaytee et al. demonstrated that a VRC-loaded nanoemulsion combined with *Pinus sylvestris* essential oil showed markedly improved antifungal activity against *Microsporum canis* compared to either VRC suspension or the essential oil alone, with inhibition zones reaching 80.3 mm, while the blank formulation showed no activity [41]. Similarly, Ashara et al. reported significant inhibition of *Saccharomyces cerevisiae* using a microemulgel containing VRC, surpassing the activity of both DMSO and a marketed ketoconazole cream Nizral^®^ [42]. El-Hadidy et al. also confirmed that MEs with VRC, especially when formulated with penetration enhancers like oleic acid or sodium deoxycholate, demonstrated superior antifungal activity against *C. albicans* compared to a saturated VRC solution, with inhibition zones up to 36 mm [30].

In line with these findings, our results showed that formulations B_2_ and B_3_ exhibited excellent antifungal activity across all three tested *C. albicans* strains, including the fluconazole-resistant clinical isolate. Notably, formulation B_4_ did not exhibit activity against the fluconazole-resistant strain. The ability of B_2_ and B_3_ to inhibit the growth of a fluconazole-resistant strain is particularly significant, as drug-resistant *Candida* infections are an increasing clinical concern and represent a major challenge in antifungal therapy.

Inhibition zones showed a general decrease after 48 h compared with 24 h. In contrast, formulation B_3_ exhibited only a negligible reduction, indicating greater stability of its inhibitory effect over time. This is a desirable characteristic for topical treatment of cutaneous mycoses and further supports the stability and integrity of the ME-based systems as drug delivery vehicles. The placebo gel, which lacked active antifungal ingredients, showed no inhibition zones, confirming that the antifungal effect was solely due to the presence of VRC (and menthol in formulation B_4_), rather than the carrier itself.

A particularly noteworthy observation was the comparison between formulation B_2_ and B_3_, which suggests a synergistic antifungal effect when menthol is co-administered with VRC. Zore et al. reported that menthol exhibits strong anti-*Candida* activity against various morphotypes and resistance phenotypes of *C. albicans*. The proposed mechanism involves disruption of membrane integrity, oxidative stress induction, cell cycle arrest, and apoptosis [43]. Samber et al. found that menthol and mint essential oil target the PM-ATPase of *Candida* species and interfere with the ergosterol biosynthesis pathway. Their reactive hydroxyl moieties also disrupt membrane stability, contributing to antifungal efficacy [44].

Moreover, Norouzi et al. demonstrated a clear synergistic interaction between menthol and VRC using checkerboard and time-kill assays, as well as anti-biofilm studies, showing enhanced effectiveness against resistant *Candida* isolates [45]. Our results corroborate these findings in an agar-based model, as evidenced by the larger inhibition zones in formulation B_3_ compared to B_2_ (*p* < 0.05) for all fungal strains tested. This highlights the potential of menthol as a valuable co-active component in antifungal formulations.

Our study not only confirms the antifungal efficacy of VRC-loaded ME gels but also supports the synergistic role of menthol as a potentiator of antifungal activity. These findings align with existing literature and suggest that the developed formulations may offer a promising alternative for the topical treatment of fungal infections, particularly in cases involving drug-resistant *Candida* strains.

The ME-based gels (B_2_ and B_3_) were evaluated for their ability to deliver VRC into full-thickness human skin. The results demonstrated that VRC penetrated the skin from both formulations, with higher deposition observed for the B_3_ gel compared with B_2_. This finding indicates an additional role of menthol as a penetration enhancer, facilitating increased drug permeation across the skin barrier, consistent with previous literature reports. Although this difference was not statistically significant (*p* = 0.1489), it should be noted that the experiments were conducted with a small sample size (n = 3), which may have substantially affected the ability to detect statistical significance.

Importantly, the amount of VRC detected in the receptor fluid after 24 h was below the analytical method’s limit of detection. This outcome aligns with the formulation’s intended design to act primarily within the skin, rather than permeating into the systemic circulation, thereby minimizing the risk of systemic adverse effects associated with VRC exposure.

A review of the available literature did not reveal any studies assessing VRC penetration and deposition in full-thickness human skin, precluding direct comparison of our findings with previously published data. Nevertheless, considering the established MIC breakpoints for VRC against *Candida* species (susceptible ≤1 μg/mL; susceptible dose dependent 2 μg/mL; resistant ≥4 μg/mL) [46], it can be stated that the VRC concentrations achieved in the skin from both B_2_ and B_3_ far exceed these thresholds. However, it must be acknowledged that tissue concentrations are expressed in μg/g of tissue, whereas MIC values are expressed in μg/mL of inoculum. Moreover, MIC values inherently refer to the unbound fraction of the drug, whereas VRC deposited in the skin may also be present in a protein-bound state.

As highlighted by Felton et al., tissue homogenates contain drug fractions distributed to various compartments (intracellular, interstitial, and vascular) and may not accurately reflect the fraction of the drug directly available to infecting microorganisms. Moreover, even when measured tissue concentrations exceed the MIC of the target pathogen, the clinical relevance of such data remains contentious [47].

In summary, comparing tissue concentrations (μg/g) with MIC values (μg/mL) is a common approach to estimate whether a drug achieves sufficient levels at the target site. However, such comparisons require explicit acknowledgment of the underlying assumptions and cautious interpretation in the context of potential clinical efficacy [48]. Thus, while the deposited levels are high, future work should include ex vivo antifungal activity in infected skin models to confirm efficacy at the target site.

## 5. Conclusions

Triacetin-based ME hydrogels were successfully formulated to deliver VRC into the skin. When gelled with Carbopol^®^, these systems remained clear and exhibited non-Newtonian, shear-thinning flow behavior and low thixotropy, which facilitates application and ensures stability. Ex vivo studies confirmed that VRC penetrated into full-thickness human skin from the gels, without detectable drug in the receptor fluid after 24 h. This indicates effective dermal delivery with minimal risk of systemic permeation. Importantly, inclusion of menthol (2%) markedly increased the amount of VRC deposited in the skin, consistent with its known role as a permeation enhancer. Microbiological testing showed potent antifungal efficacy of the ME-hydrogels: VRC-loaded formulations produced clear inhibition zones against *C. albicans* (including a fluconazole-resistant strain). The gel with VRC and menthol consistently gave larger zones than VRC alone, confirming a synergistic benefit. Overall, the developed ME-hydrogels combine enhanced VRC skin delivery, improved deposition with menthol, favorable rheological properties, and strong activity against tested *Candida* spp. These features demonstrate their promise as advanced topical treatments for fungal skin infections.

## Figures and Tables

**Table 1:** Composition of tested systems used for pseudoternary phase diagram construction. Each formulation consists of an oil phase, surfactant (S), co-surfactant (CoS), and a specified S:CoS weight ratio.

Sample Name	Oil Phase	Surfactant (S)	Co-Surfactant (CoS)	S:CoS Weight Ratio
A_2.1_	Triacetin	Tween 80	Transcutol	1:1
A_4.1_	Triacetin	Etocas 35	Transcutol	1:1
A_9.1_	Triacetin	Brij O5-SS-(RB)	Transcutol	1:1
A_13.1_	Isopropyl palmitate	Brij O5-SS-(RB)	Transcutol	1:1
A_16.1_	Ethyl oleate	Brij O5-SS-(RB)	Transcutol	1:1
A_19.1_	Oleic acid	Brij O5-SS-(RB)	Transcutol	1:1
A_23.1_	Isopropyl myristate	Brij O5-SS-(RB)	Transcutol	1:1

**Table 2:** Composition of VRC-loaded ME-based hydrogels and placebo formulation (percent *w*/*w*). B_2_–B_4_: test formulations; Placebo: drug-free control. “x” indicates absence of the ingredient. For pH analysis, the results are presented as mean ± standard deviation (SD), n = 3.

Ingredient	B_2_	B_3_	B_4_	Placebo
Voriconazole	0.5	0.5	x	x
Triacetin	8.76	8.46	8.61	8.91
Transcutol^®^	10.22	9.87	10.045	10.395
Etocas^TM^ 35	10.22	9.87	10.045	10.395
Water	19.0	19.0	19.0	19.0
Carbopol^®^	1.0	1.0	1.0	1.0
TIPA	0.3	0.3	0.3	0.3
Menthol	x	1.0	1.0	x
pH	5.42 ± 0.01	5.35 ± 0.01	5.47 ± 0.01	5.49 ± 0.01

**Table 3:** Solubility of voriconazole [mg/mL] in selected oils, surfactants, and co-surfactant at 25 °C and 32 °C. “x” indicates that solubility could not be determined due to methodological limitations. Solubility in Tween^®^ 80 was not experimentally determined; thus, in the table, the literature values are provided.

Medium	Solubility [mg/mL] at Temperature	
	25 °C	32 °C
Triacetin	87.88	117.7
Oleic Acid	43.09	52.19
Ethyl Oleate	6.37	8.52
Isopropyl Palmitate	5.13	6.89
Isopropyl Myristate	5.83	8.01
Tween^®^ 80	26.86 (37 °C) [19]	
Etocas^TM^ 35	x	x
Brij^®^ O5-SS-(RB)	23.82	31.1
Transcutol^®^	173.93	202.36

**Table 4:** Compositions of the A_4.1_ microemulsions containing voriconazole and/or menthol (+placebo). “x” indicates absence of the ingredient.

Ingredient [g]	1% VRC	1% VRC + 2% Menthol	2% Menthol	Placebo
VRC	1.0	1.0	x	x
Triacetin	18.3	17.7	18.0	18.6
Transcutol^®^	21.35	20.65	21.0	21.7
Etocas^TM^ 35	21.35	20.65	21.0	21.7
Water	38.0	38.0	38.0	38.0
Menthol	x	2.0	2.0	x

**Table 5:** Physicochemical parameters of the A_4.1_ microemulsions containing voriconazole and/or menthol (+placebo). Results are presented as mean ± standard deviation (SD), n = 3.

Parameter	1% VRC	1% VRC + 2% Menthol	2% Menthol	Placebo
pH	5.183 ± 0.006	5.17 ± 0.01	5.18 ± 0.00	5.23 ± 0.00
Viscosity [mPa·s]	41.423 ± 0.046	40.41 ± 0.024	40.685 ± 0.021	41.618 ± 0.029
Size [nm]	2.822 ± 0.052	2.962 ± 0.034	2.965 ± 0.116	2.917 ± 0.100
PDI	0.439 ± 0.019	0.454 ± 0.030	0.467 ± 0.018	0.476 ± 0.025
RI	1.4073	1.4083	1.4078	1.4074

**Table 6:** Drug content of microemulsion-based hydrogels. Results are presented as mean ± standard deviation (SD), n = 3.

Formulation	Drug Content [%]
B_2_	0.996 ± 0.023
B_3_	1.021 ± 0.021

**Table 7:** Rheological parameters obtained from controlled shear rate (CR) and controlled stress (CS) flow curve analyses. Kd—thixotropic breakdown coefficient. Results are presented as mean ± standard deviation (SD), n = 3.

Parameter	B_2_	B_3_	B_4_	Placebo
*τ*_0_ [Pa]	140.17 ± 8.30	117.50 ± 13.93	144.73 ± 14.59	148.00 ± 3.17
K [Pa*·s^n^]	41.25 ± 4.83	45.47 ± 5.93	36.63 ± 1.14	31.43 ± 0.47
n [−]	0.5698 ± 0.0231	0.545 ± 0.0246	0.5754 ± 0.0074	0.5874 ± 0.0046
Kd [%]	8.91 ± 0.11	8.52 ± 0.15	7.76 ± 0.30	7.78 ± 0.14
yield point [Pa]	72.80 ± 1.01	67.90 ± 0.80	69.83 ± 0.70	68.76 ± 1.63

**Table 8:** The parameter values determined during the spreadability test are presented as the mean of three measurements (n = 3) ± SD.

Formulation	Adhesiveness [mJ]	Firmness [N]	Spreadability [mJ]	Adhesion Force [N]
B_2_	−0.5916 ± 0.0726	0.3142 ± 0.0212	0.6016 ± 0.0250	−0.1400 ± 0.0030
B_3_	−0.7110 ± 0.0252	0.3454 ± 0.0203	0.6560 ± 0.0222	−0.1685 ± 0.0131
B_4_	−0.6279 ± 0.0620	0.2959 ± 0.0377	0.5696 ± 0.0455	−0.1355 ± 0.0227
P	−0.6273 ± 0.0786	0.2780 ± 0.0372	0.5416 ± 0.0620	−0.1367 ± 0.0232

Adhesiveness refers to the work required to withdraw the probe from the sample, while firmness corresponds to the maximum force recorded during probe penetration. Springiness denotes the force registered throughout the probe penetration process, whereas adhesion force is defined as the minimum force value recorded.

## Data Availability

The data presented in this study are available on request from the corresponding author.

## Supplementary Materials

The following supporting information can be downloaded at: https://www.mdpi.com/article/10.3390/pharmaceutics17091218/s1. Table S1: Composition of tested systems used for pseudoternary phase diagram construction. Each formulation consists of an oil phase, surfactant (S), co-surfactant (CoS), and a specified S:CoS weight ratio. Table S2: Summary of validation parameters for HPLC-UV methods of VRC determination in various media. Table S3: Quantitative composition of ME formulations A_1_–A_23_ (amounts of oil phase, surfactant/co-surfactant, and water in grams). Figure S1: Electrical conductivity [μS·cm^−1^] as a function of 0.01% NaCl phase content [%, *w*/*w*] for system A_9.1_ at oil/S:CoS ratios of 4:6, 3:7, and 2:8. The blue dotted lines indicate the approximate structural transition points. Figure S2: Electrical conductivity [μS·cm^−1^] as a function of 0.01% NaCl phase content [%, *w*/*w*] for system A_13.1_ at oil/S:CoS ratios of 4:6, 3:7, and 2:8. The blue dotted lines indicate the approximate structural transition points. Figure S3: Electrical conductivity [μS·cm^−1^] as a function of 0.01% NaCl phase content [%, *w*/*w*] for system A_16.1_ at oil/S:CoS ratios of 4:6, 3:7, and 2:8. The blue dotted lines indicate the approximate structural transition points. Figure S4: Electrical conductivity [μS·cm^−1^] as a function of 0.01% NaCl phase content [%, *w*/*w*] for system A_19.1_ at oil/S:CoS ratios of 4:6, 3:7, and 2:8. The blue dotted lines indicate the approximate structural transition points. Figure S5: Electrical conductivity [μS·cm^−1^] as a function of 0.01% NaCl phase content [%, *w*/*w*] for system A_23.1_ at oil/S:CoS ratios of 4:6, 3:7, and 2:8. The blue dotted lines indicate the approximate structural transition points.

## Abbreviations

The following abbreviations are used in this manuscript:

VRC	Voriconazole
ME	Microemulsion
PDI	Polydispersity Index
WHO	World Health Organization
R&D	Research and Development
API	Active Pharmaceutical Ingredient
PBS	Phosphate-Buffered Saline
DLS	Dynamic Light Scattering
HPLC	High-Performance Liquid Chromatography
LOD	Limit of Detection
LOQ	Limit of Quantification
TIPA	Triisopropanolamine
CFU	Colony Forming Units
S:CoS	Surfactant-to-Co-surfactant Ratio
w/o	Water-in-Oil (emulsion type)
o/w	Oil-in-Water (emulsion type)
RI	Refractive Index
ME-hydrogels	Microemulsion-based Hydrogels
CR	Controlled Rate (in rheological measurements)
CS	Controlled Stress (in rheological measurements)
EO	Ethylene Oxide
SC	Stratum Corneum
HLB	Hydrophilic-Lipophilic Balance
TPA	Texture Profile Analysis
