# Supplementary material for: Formulation Studies on Microemulsion-Based Polymer Gels Loaded with Voriconazole for the Treatment of Skin Mycoses
Source: Pharmaceutics. 2025 Sep 18;17(9):1218. doi: 10.3390/pharmaceutics17091218 (PMC12473683; doi:10.3390/pharmaceutics17091218)
Supplement: Supplementary file 1 [file pharmaceutics-17-01218-s001.zip › pharmaceutics-3869113-supplementary.pdf]

Supplementary Materials

Table S1 Composition of tested systems used for pseudoternary phase diagram construction. Each formulation consists of an oil phase, surfactant (S), co-surfactant (CoS), and a specified S:CoS weight ratio.

Sample name	Oil phase	Surfactant (S)	Co-surfactant (CoS)	S;CoS weight ratio
$A_{1.1}$	Triacetin	Tween 80	PEG 400	1:1
$A_{1.2}$	Triacetin	Tween 80	PEG 400	1:2
$A_{2.1}$	Triacetin	Tween 80	Transcutol	1:1
$A_{2.2}$	Triacetin	Tween 80	Transcutol	1:2
$A_{3.1}$	Triacetin	Etocas 35	PEG 400	1:1
$A_{3.2}$	Triacetin	Etocas 35	PEG 400	1:2
$A_{4.1}$	Triacetin	Etocas 35	Transcutol	1:1
$A_{4.2}$	Triacetin	Etocas 35	Transcutol	1:2
$A_{5.1}$	Neobee M5	Tween 80	PEG 400	1:1
$A_{5.2}$	Neobee M5	Tween 80	PEG 400	1:2
$A_{6.1}$	Neobee M5	Tween 80	Transcutol	1:1
$A_{6.2}$	Neobee M5	Tween 80	Transcutol	1:2
$A_{7.1}$	Neobee M5	Etocas 35	PEG 400	1:1
$A_{7.2}$	Neobee M5	Etocas 35	PEG 400	1:2
$A_{8.1}$	Neobee M5	Etocas 35	Transcutol	1:1
$A_{8.2}$	Neobee M5	Etocas 35	Transcutol	1:2
$A_{9.1}$	Triacetin	Brij O5-SS-(RB)	Transcutol	1:1
$A_{9.2}$	Triacetin	Brij O5-SS-(RB)	Transcutol	1:2
$A_{10.1}$	Triacetin	Ludox HS-30 coll.	Transcutol	1:1
$A_{10.2}$	Triacetin	Ludox HS-30 coll.	Transcutol	1:2
$A_{11.1}$	Isopropyl palmitate	Tween 80	Transcutol	1:1
$A_{11.2}$	Isopropyl palmitate	Tween 80	Transcutol	1:2
$A_{12.1}$	Isopropyl palmitate	Etocas 35	Transcutol	1:1
$A_{12.2}$	Isopropyl palmitate	Etocas 35	Transcutol	1:2
$A_{13.1}$	Isopropyl palmitate	Brij O5-SS-(RB)	Transcutol	1:1
$A_{13.2}$	Isopropyl palmitate	Brij O5-SS-(RB)	Transcutol	1:2
$A_{14.1}$	Ethyl oleate	Tween 80	Transcutol	1:1
$A_{14.2}$	Ethyl oleate	Tween 80	Transcutol	1:2
$A_{15.1}$	Ethyl oleate	Etocas 35	Transcutol	1:1
$A_{15.2}$	Ethyl oleate	Etocas 35	Transcutol	1:2
$A_{16.1}$	Ethyl oleate	Brij O5-SS-(RB)	Transcutol	1:1
$A_{16.2}$	Ethyl oleate	Brij O5-SS-(RB)	Transcutol	1:2
$A_{17.1}$	Oleic acid	Tween 80	Transcutol	1:1
$A_{17.2}$	Oleic acid	Tween 80	Transcutol	1:2
$A_{18.1}$	Oleic acid	Etocas 35	Transcutol	1:1
$A_{18.2}$	Oleic acid	Etocas 35	Transcutol	1:2
$A_{19.1}$	Oleic acid	Brij O5-SS-(RB)	Transcutol	1:1
$A_{19.2}$	Oleic acid	Brij O5-SS-(RB)	Transcutol	1:2
$A_{20.1}$	Triacetin:oleic acid (3:1)	Tween 80	Transcutol	1:1
$A_{20.2}$	Triacetin:oleic acid (3:1)	Tween 80	Transcutol	1:2
$A_{21.1}$	Oleic acid	Tween 85	Transcutol	1:1
$A_{21.2}$	Oleic acid	Tween 85	Transcutol	1:2
$A_{22.1}$	Isopropyl myristate	Etocas 35	Transcutol	1:1
$A_{22.2}$	Isopropyl myristate	Etocas 35	Transcutol	1:2
$A_{23.1}$	Isopropyl myristate	Brij O5-SS-(RB)	Transcutol	1:1
$A_{23.2}$	Isopropyl myristate	Brij O5-SS-(RB)	Transcutol	1:2

Table S2 Summary of validation parameters for HPLC - UV methods of VRC determination in various media.

Concentration range for the method: 0.1–200 µg/mL.

Validation parameter	PBS (pH = 7.4)	Ethanol absolute
Specifity	Yes	Yes
Linearity	y=12535.33x+13464.67	y=12421x+7997.33
Limit of detection (LOD) [a)]	4.66 ± 0.63 µg/mL	5.68 ± 1.12 µg/mL
Limit of quantification [b)]	14.13 ± 1.92 µg/mL	17.20 ± 3.41 µg/mL
Accuracy (%) [c)]	25 µg/mL : 7.10 ± 0.69<br>100 µg/mL : 2.57 ± 0.68<br>200 µg/mL : 0.55 ± 0.03	25 µg/mL : 4.59 ± 4.56<br>100 µg/mL : 1.27 ± 0.91<br>200 µg/mL : 1.19 ± 0.95
Precision (%) [d)]	25 µg/mL : 0.08 ± 0.06<br>100 µg/mL : 0.10 ± 0.06<br>200 µg/mL : 0.04 ± 0.03	25 µg/mL : 0.32 ± 0.14<br>100 µg/mL : 0.16 ± 0.14<br>200 µg/mL : 0.12 ± 0.13

[a)] Calculated based on the Standard Error; using the formula: LOD = 3.3 × (SE of intercept / slope);

[b)] Calculated based on the Standard Error; using the formula: LOQ = 10 × (SE of intercept / slope);

[c)] Expressed as the error of determination in % = |(mean determined concentration − nominal concentration) / (nominal concentration)| × 100%

[d)] Expressed as coefficient of variation CV [%] = (standard deviation of determined concentrations) / (mean determined concentration) × 100%

Table S3 Quantitative composition of ME formulations A1–A23 (amounts of oil phase. surfactant:co-surfactant. and water in grams).

**$A_{1.1}$**		
Oli phase [g]	S:CoS [g]	H_2O [g]
1.81	0.21	0.1
1.59	0.42	0.15
1.42	0.59	0.15
1.2	0.83	0.2
1.09	1.02	0.2
0.8	1.25	0.2
0.6	1.42	0.3
0.41	1.59	0.65
0.21	1.79	∞
**$A_{1.2}$**		
Oli phase [g]	S:CoS [g]	H_2O [g]
1.8	0.2	0.1
1.64	0.41	0.15
1.38	0.67	0.2
1.2	0.83	0.25
1.01	1.01	0.25
0.79	1.23	0.2
0.61	1.4	0.1
0.41	1.6	0.05
0.2	1.8	0.05
**$A_{2.1}$**		
Oli phase [g]	S:CoS [g]	H_2O [g]
1.83	0.21	0.15
1.63	0.4	0.15
1.41	0.61	0.2
1.2	0.8	0.35
0.98	1	0.6
0.84	1.19	0.85
0.58	1.41	1.7
0.42	1.63	∞
0.21	1.82	∞
**$A_{2.2}$**		
Oli phase [g]	S:CoS [g]	H_2O [g]
1.8	0.2	0.12
1.62	0.46	0.18
1.39	0.57	0.26
1.18	0.83	0.55
1	1	0.8
0.77	1.22	1.15
0.59	1.41	2.2
0.41	1.59	∞
0.19	1.85	∞
**$A_{3.1}$**		
Oli phase [g]	S:CoS [g]	H_2O [g]
1.83	0.22	0.05

**$A_{12.2}$**		
Oli phase [g]	S:CoS [g]	H_2O [g]
1.59	0.49	0.02
1.2	0.79	0.02
1.03	1	0.02
0.83	1.23	0.05
0.6	1.39	0.15
0.43	1.61	0.15
**$A_{13.1}$**		
Oli phase [g]	S:CoS [g]	H_2O [g]
1.58	0.43	0.06
1.19	0.79	0.2
1.04	1.03	1.2
0.81	1.28	1.2
0.62	1.49	0.8
0.41	1.63	0.8
**$A_{13.2}$**		
Oli phase [g]	S:CoS [g]	H_2O [g]
1.64	0.44	0.02
1.21	0.92	0.15
1.05	1.06	0.2
0.83	1.23	0.4
0.63	1.41	1.6
0.62	1.88	1.6
**$A_{14.1}$**		
Oli phase [g]	S:CoS [g]	H_2O [g]
1.62	0.5	0.04
1.19	0.85	0.04
1.02	1.04	0.06
0.77	1.2	0.1
0.62	1.4	0.2
0.44	1.64	0.4
**$A_{14.2}$**		
Oli phase [g]	S:CoS [g]	H_2O [g]
1.62	0.44	0.02
1.21	0.85	0.04
1.01	1.06	0.04
0.82	1.24	0.1
0.63	1.53	0.15
0.41	1.61	0.2
**$A_{15.1}$**		
Oli phase [g]	S:CoS [g]	H_2O [g]
1.59	0.39	0.04
1.2	0.81	0.06
1	1.03	0.06
0.83	1.23	0.1
0.6	1.42	0.3

1.63	0.41	0.1	0.42	1.62	0.8
1.42	0.6	0.15	**$A_{15.2}$**		
1.22	0.84	0.15	Oli phase [g]	S:CoS [g]	H_2O [g]
1	1.02	0.15	1.91	0.74	0.02
0.81	1.19	0.2	1.27	0.85	0.04
0.61	1.4	0.2	1.02	0.97	0.04
0.42	1.62	∞	0.86	1.22	0.08
0.21	1.86	∞	0.61	1.41	0.1
**$A_{3.2}$**			0.43	1.73	0.3
Oli phase [g]	S:CoS [g]	H_2O [g]	**$A_{16.1}$**		
1.63	0.39	0.05	Oli phase [g]	S:CoS [g]	H_2O [g]
1.23	0.81	0.05	1.66	0.45	0.1
0.79	1.22	0.05	1.24	0.81	0.25
0.6	1.41	0.05	1.02	1.02	0.75
0.41	1.58	0.05	0.83	1.25	1.3
**$A_{4.1}$**			0.68	1.41	1.6
Oli phase [g]	S:CoS [g]	H_2O [g]	0.43	1.61	2.4
1.79	0.21	0.07	**$A_{16.2}$**		
1.62	0.46	0.15	Oli phase [g]	S:CoS [g]	H_2O [g]
1.45	0.65	0.2	1.61	0.45	0.05
1.24	0.88	0.45	1.21	0.84	0.15
0.99	1.02	0.6	1.01	1.04	0.25
0.82	1.19	0.85	0.78	1.24	1.6
0.64	1.47	1.7	0.62	1.42	0.85
0.42	1.63	∞	0.44	1.63	1.4
0.21	1.81	∞	**$A_{17.1}$**		
**$A_{4.2}$**			Oli phase [g]	S:CoS [g]	H_2O [g]
Oli phase [g]	S:CoS [g]	H_2O [g]	1.6	0.54	0.15
1.82	0.25	0.1	1.21	0.78	0.2
1.58	0.45	0.15	1.23	1.28	0.3
1.41	0.61	0.25	0.88	1.34	0.35
1.25	0.79	0.5	0.61	1.43	1
0.98	0.97	0.9	0.42	1.59	0.9
0.78	1.25	1.35	**$A_{17.2}$**		
0.62	1.45	∞	Oli phase [g]	S:CoS [g]	H_2O [g]
0.4	1.61	∞	1.65	0.55	0.1
0.26	1.86	∞	1.23	0.83	0.2
**$A_{5.1}$**			1	1.02	0.3
Oli phase [g]	S:CoS [g]	H_2O [g]	0.81	1.24	0.4
1.82	0.19	0	0.63	1.42	0.55
1.57	0.39	0	0.43	1.71	1.6
1.38	0.6	0	**$A_{18.1}$**		
1.24	0.86	0	Oli phase [g]	S:CoS [g]	H_2O [g]
0.98	1	0	1.64	0.41	0.1
0.78	1.18	0	1.21	0.79	0.2
0.59	1.41	0	1	0.99	0.25
0.39	1.62	0	0.8	1.25	0.35
0.2	1.83	0	0.61	1.42	0.45
**$A_{5.2}$**			0.43	1.64	1

Oli phase [g]	S:CoS [g]	H_2O [g]
The sample was excluded based on the outcome of test A5.1		
**$A_{6.1}$**		
Oli phase [g]	S:CoS [g]	H_2O [g]
1.6	0.42	0.05
1.01	1.06	0.1
0.85	1.19	0.1
0.61	1.41	0.1
0.42	1.67	0.15
**$A_{6.2}$**		
Oli phase [g]	S:CoS [g]	H_2O [g]
The sample was excluded based on the outcome of test A6.1		
**$A_{7.1}$**		
Oli phase [g]	S:CoS [g]	H_2O [g]
1.6	0.49	0.05
0.99	1.01	1.01
0.87	1.27	1.27
0.61	1.38	1.38
0.41	1.61	1.61
**$A_{7.2}$**		
Oli phase [g]	S:CoS [g]	H_2O [g]
The sample was excluded based on the outcome of test A7.1		
**$A_{8.1}$**		
Oli phase [g]	S:CoS [g]	H_2O [g]
The sample was excluded based on the previous outcomes.		
**$A_{8.2}$**		
Oli phase [g]	S:CoS [g]	H_2O [g]
The sample was excluded based on the previous outcomes.		
**$A_{9.1}$**		
Oli phase [g]	S:CoS [g]	H_2O [g]
1.8	0.26	0.14
1.58	0.53	0.25
1.48	0.74	0.4
1.21	0.9	0.4
1.09	1.08	0.55
0.9	1.29	0.8
0.64	1.43	1.3
0.44	1.71	3.25
0.22	1.86	3.3

**$A_{18.2}$**		
Oli phase [g]	S:CoS [g]	H_2O [g]
0.63	0.5	0.1
1.19	0.89	0.25
1	1.01	0.3
0.8	1.24	0.4
0.63	1.47	0.55
0.42	1.68	0.9
**$A_{19.1}$**		
Oli phase [g]	S:CoS [g]	H_2O [g]
1.59	0.41	0.05
1.2	0.78	0.2
1.01	1.02	0.3
0.81	1.25	0.5
0.62	1.37	0.75
0.39	1.58	0.8
**$A_{19.2}$**		
Oli phase [g]	S:CoS [g]	H_2O [g]
1.68	0.45	0.05
1.21	0.79	0.2
1.02	1.01	0.35
0.78	1.21	0.5
0.6	1.41	0.7
0.41	1.63	0.9
**$A_{20.1}$**		
Oli phase [g]	S:CoS [g]	H_2O [g]
1.61	0.42	0.15
1.21	0.86	0.35
1.01	0.97	0.55
0.79	1.3	1.35
0.6	1.39	3.3
0.42	1.65	∞
**$A_{20.2}$**		
Oli phase [g]	S:CoS [g]	H_2O [g]
1.6	0.38	0.15
1.25	0.85	0.45
1.1	1.03	0.7
0.88	1.22	1.25
0.66	1.43	3.1
0.42	1.62	2.7
**$A_{21.1}$**		
Oli phase [g]	S:CoS [g]	H_2O [g]
1.69	0.54	0.05
1.18	0.78	0.05
0.99	0.99	0.15
0.8	1.21	0.25
0.61	1.38	0.3
0.56	1.88	0.35
**$A_{21.2}$**		

**$A_{9.2}$**		
Oli phase [g]	S:CoS [g]	H_2O [g]
1.82	0.33	0.2
1.62	0.59	0.3
1.42	0.68	0.4
1.27	0.77	0.5
1.04	1.07	0.85
0.81	1.37	1.45
0.66	1.53	4.8
0.47	1.6	4.1
0.27	1.8	3.5
**$A_{10.1}$**		
Oli phase [g]	S:CoS [g]	H_2O [g]
The formulation was excluded due to incompatibility between the surfactant and the co-surfactant.		
**$A_{10.2}$**		
Oli phase [g]	S:CoS [g]	H_2O [g]
The formulation was excluded due to incompatibility between the surfactant and the co-surfactant.		
**$A_{11.1}$**		
Oli phase [g]	S:CoS [g]	H_2O [g]
1.4	0.62	0.02
1.2	0.85	0.02
1.01	1.04	0.02
0.84	1.24	0.02
0.64	1.44	0.26
0.43	1.6	0.3
0.21	1.8	1
**$A_{11.2}$**		
Oli phase [g]	S:CoS [g]	H_2O [g]
1.62	0.46	0.02
1.2	0.78	0.02
0.99	1.02	0.02
**$A_{12.1}$**		
Oli phase [g]	S:CoS [g]	H_2O [g]
1.62	0.42	0.01
1.21	0.79	0.02
0.98	1.03	0.02
0.82	1.29	0.05
0.61	1.45	0.1
0.44	1.62	0.3
Oli phase [g]	S:CoS [g]	H_2O [g]
1.59	0.45	0.05
1.2	0.82	0.1
1	1.03	0.25
0.9	1.21	0.35
0.59	1.44	0.4
0.41	1.64	0.45
**$A_{22.1}$**		
Oli phase [g]	S:CoS [g]	H_2O [g]
1	1.02	0.03
0.83	1.24	0.06
0.62	1.39	0.15
0.43	1.59	0.5
**$A_{22.2}$**		
Oli phase [g]	S:CoS [g]	H_2O [g]
1.02	1.01	0.03
0.82	1.18	0.06
0.61	1.42	0.18
0.4	1.62	0.25
**$A_{23.1}$**		
Oli phase [g]	S:CoS [g]	H_2O [g]
1.58	0.4	0.05
1.21	0.84	0.2
1	1.02	1.1
0.81	1.19	1.5
0.66	1.51	2.6
0.39	1.6	3
**$A_{23.2}$**		
Oli phase [g]	S:CoS [g]	H_2O [g]
1.62	0.42	0.05
1.38	0.96	0.15
1	1.01	0.2
0.81	1.24	1.5
0.59	1.4	1.6
0.39	1.63	1.8

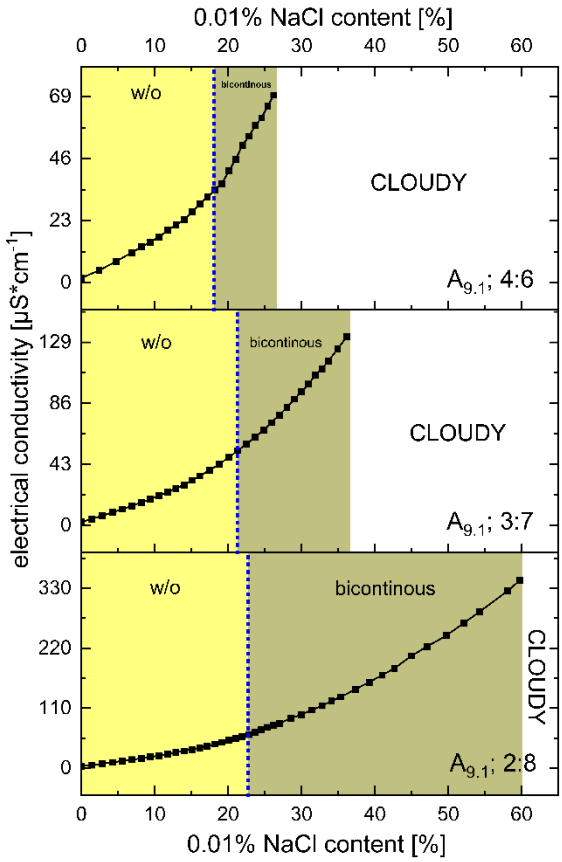

Figure S1 Electrical conductivity [µS·cm⁻¹] as a function of 0.01% NaCl phase content [%, w/w] for system $A_{9.1}$ at oil:S:CoS ratios of 4:6, 3:7, and 2:8. The blue dotted lines indicate the approximate structural transition points.

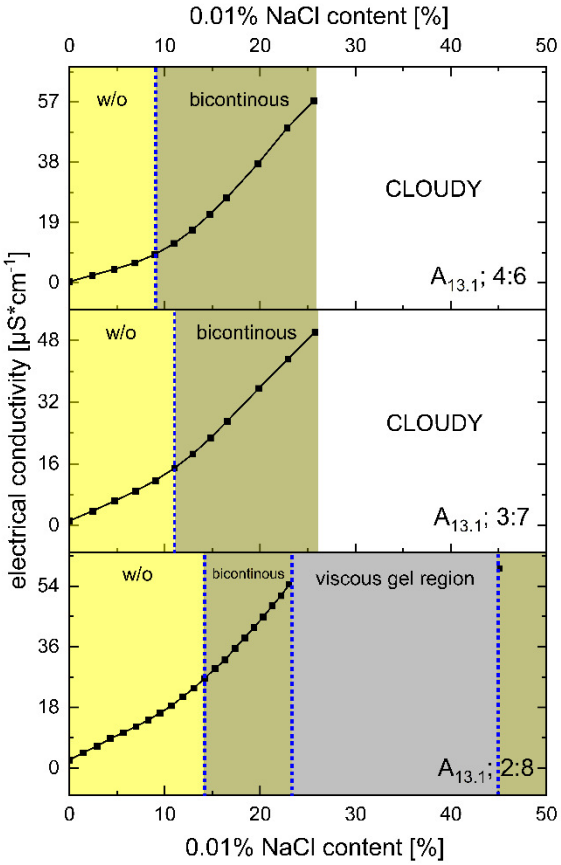

Figure S2 Electrical conductivity [µS·cm⁻¹] as a function of 0.01% NaCl phase content [%, w/w] for system $A_{13.1}$ at oil:S:CoS ratios of 4:6, 3:7, and 2:8. The blue dotted lines indicate the approximate structural transition points.

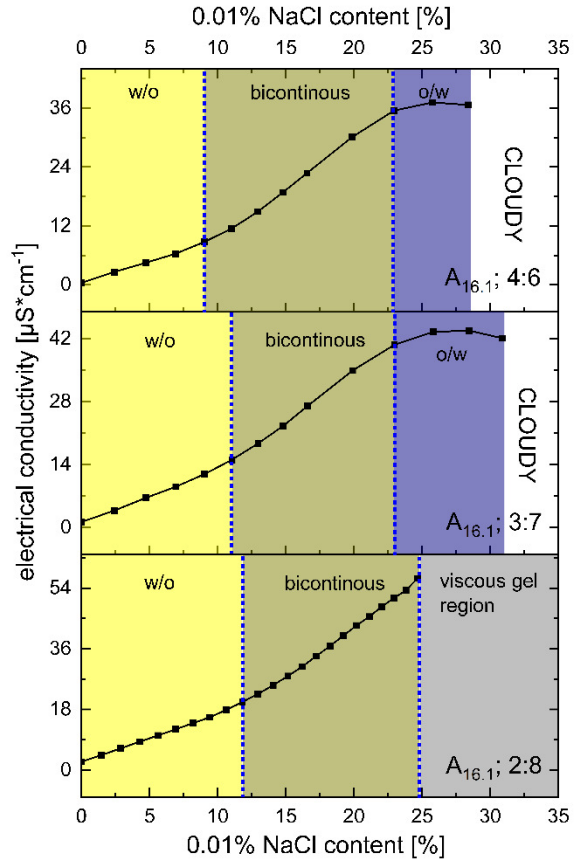

Figure S3 Electrical conductivity [µS·cm⁻¹] as a function of 0.01% NaCl phase content [%, w/w] for system $A_{16.1}$ at oil:S:CoS ratios of 4:6, 3:7, and 2:8. The blue dotted lines indicate the approximate structural transition points.

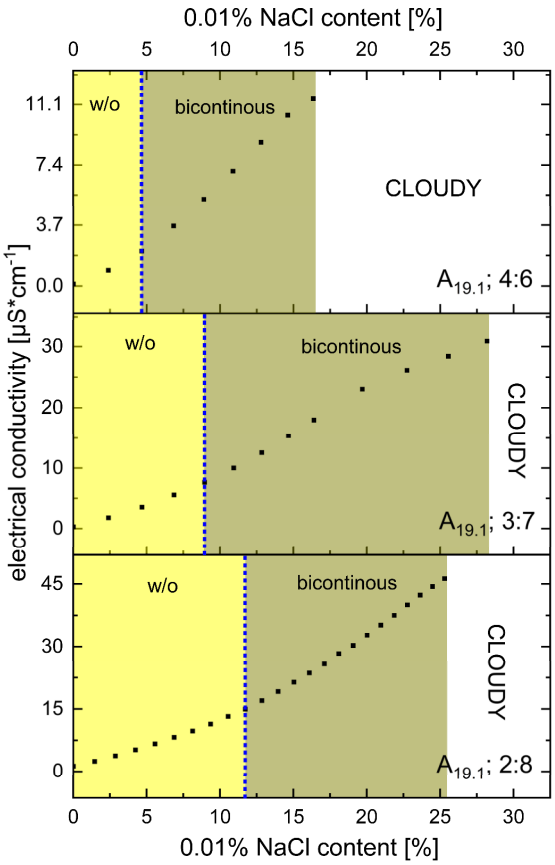

Figure S4 Electrical conductivity [µS·cm⁻¹] as a function of 0.01% NaCl phase content [%, w/w] for system $A_{19.1}$ at oil:S:CoS ratios of 4:6, 3:7, and 2:8. The blue dotted lines indicate the approximate structural transition points.

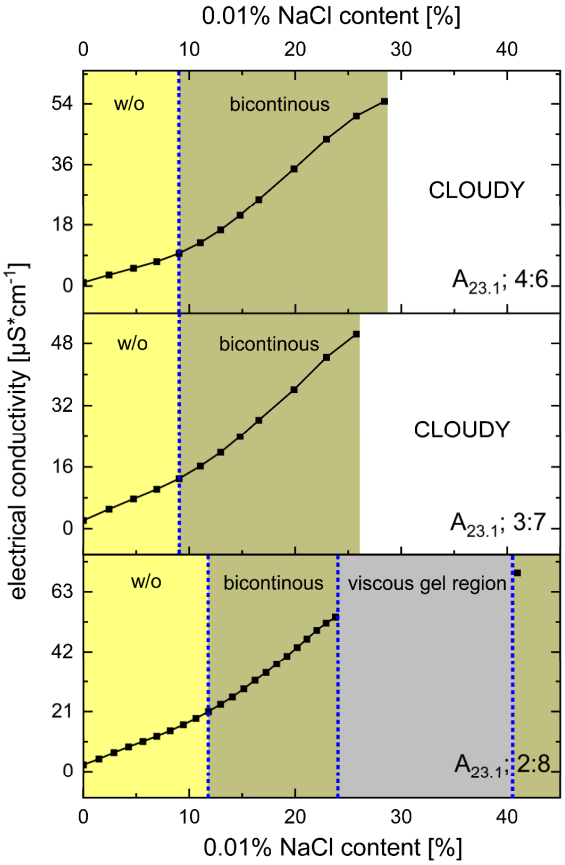

Figure S5 Electrical conductivity [µS·cm⁻¹] as a function of 0.01% NaCl phase content [%, w/w] for system $A_{23.1}$ at oil:S:CoS ratios of 4:6, 3:7, and 2:8. The blue dotted lines indicate the approximate structural transition points.
